# Bilobate leaves of *Bauhinia* (Leguminosae, Caesalpinioideae, Cercideae) from the middle Miocene of Fujian Province, southeastern China and their biogeographic implications

**DOI:** 10.1186/s12862-015-0540-9

**Published:** 2015-11-16

**Authors:** Yanxiang Lin, William Oki Wong, Gongle Shi, Si Shen, Zhenyu Li

**Affiliations:** State Key Laboratory of Systematic and Evolutionary Botany, Institute of Botany, Chinese Academy of Sciences, Beijing, 100093 P.R. China; University of Chinese Academy of Sciences, Beijing, 100049 P.R. China; State Key Laboratory of Palaeobiology and Stratigraphy, Nanjing Institute of Geology and Palaeontology, Chinese Academy of Sciences, 39 East Beijing Road, Nanjing, 210008 P.R. China

**Keywords:** *Bauhinia*, Bauhiniinae, Bilobate leaves, Biogeography, Caesalpinioideae, Cercideae, Evolution, Fotan Group, Legumes, Leguminosae, Miocene, North Atlantic Land Bridge, Orchid trees, Pantropical intercontinental disjunct, South China, Tethys Seaway

## Abstract

**Background:**

Morphological and molecular phylogenetic studies suggest that the pantropical genus *Bauhinia* L. s.l. (Bauhiniinae, Cercideae, Leguminosae) is paraphyletic and may as well be subdivided into nine genera, including *Bauhinia* L. s.s. and its allies. Their leaves are usually characteristic bilobate and are thus easily recognized in the fossil record. This provides the opportunity to understand the early evolution, diversification, and biogeographic history of orchid trees from an historical perspective under the framework of morphological and molecular studies.

**Results:**

The taxonomy, distribution, and leaf architecture of *Bauhinia* and its allies across the world are summarized in detail, which formed the basis for classifying the bilobate leaf fossils and evaluating the fossil record and biogeography of *Bauhinia*. Two species of *Bauhinia* are described from the middle Miocene Fotan Group of Fujian Province, southeastern China. *Bauhinia ungulatoides* sp. nov. is characterized by shallowly to moderately bilobate, pulvinate leaves with shallowly cordate bases and acute apices on each lobe, as well as paracytic stomatal complexes. *Bauhinia fotana* F.M.B. Jacques et al. emend. possesses moderately bilobate, pulvinate leaves with moderately to deeply cordate bases and acute or slightly obtuse apices on each lobe.

**Conclusions:**

Bilobate leaf fossils *Bauhinia ungulatoides* and *B. fotana* together with other late Paleogene – early Neogene Chinese record of the genus suggest that *Bauhinia* had been diverse in South China by the late Paleogene. Their great similarities to some species from South America and South Asia respectively imply that *Bauhinia* might have undergone extensive dispersals and diversification during or before the Miocene. The fossil record, extant species diversity, as well as molecular phylogenetic analyses demonstrate that the Bauhiniinae might have originated in the Paleogene of low-latitudes along the eastern Tethys Seaway. They dispersed southwards into Africa, migrated from Eurasia to North America via the North Atlantic Land Bridge or floating islands during the Oligocene. Then the genus spread into South America probably via the Isthmus of Panama since the Miocene onward, and underwent regional extinctions in the Boreotropics of mid-high-latitudes during the Neogene climatic cooling. Hence, *Bauhinia* presently exhibits a pantropical intercontinental disjunct distribution.

**Electronic supplementary material:**

The online version of this article (doi:10.1186/s12862-015-0540-9) contains supplementary material, which is available to authorized users.

## Background

**Fig. 1:**
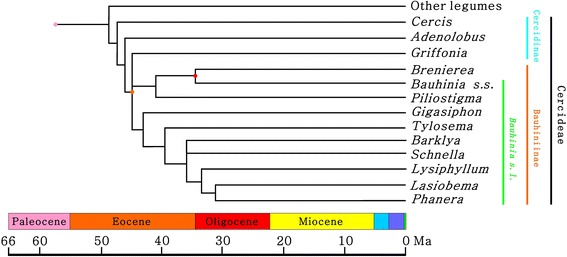
A simplified diagram showing the phylogenetic relationships and divergence times of *Bauhinia* L. s.s. and its allies (after [12, 13]). Nodes of the cladogram refer to key leaf fossils [28–31] and the latest International Chronostratigraphic Chart [58]

Leguminosae Juss. are the third largest angiosperm family with various growth habits across different habitats of the world, including about 751 extant genera and ca. 19,500 species [1–3]. Traditionally, the family is divided into three subfamilies, i.e., Caesalpinioideae DC., Mimosoideae DC., and Papilionoideae L. ex DC. [4, 5]. Advances in legume systematics during recent decades, however, have challenged this traditional classification scheme and also the circumscription of some large legume genera [1, 3]. *Bauhinia* L. s.l. (commonly known as the orchid tree), being a pantropical large genus with 340 species in the tribe Cercideae Bronn, is among the legumes with such a problem. Historical factors have complicated the taxonomy and nomenclature of *Bauhinia* [6–11]. Recent molecular phylogenetic and palynological studies have revealed that *Bauhinia* L. s.l. is not monophyletic [2, 12–14] and may as well be split into nine separate genera, including *Bauhinia* L. s.s., *Barklya* F. Muell., *Gigasiphon* Drake, *Lasiobema* (Korth.) Miq., *Lysiphyllum* (Benth.) de Wit, *Phanera* Lour., *Piliostigma* Hochst., *Schnella* Raddi, and *Tylosema* (Schweinf.) Torre et Hillc. [6–11, 15–22] (Fig. 1; Table 1; see Additional file 1). We adopt this classification scheme, and these genera are referred to as “*Bauhinia* and its allies” in the present study.

**Table 1:** A comparison of habit, floral part, distribution and species diversity between *Bauhinia* and its allies [9–11, 15–22]

Taxon name	*Bauhinia* L. s.s.	*Barklya* F. Muell.	*Gigasiphon* Drake	*Lasiobema* (Korth.) Miq.	*Lysiphyllum* (Benth.) de Wit	*Phanera* Lour.	*Piliostigma* Hochst.	*Schnella* Raddi	*Tylosema* (Schweinf.) Torre et Hillc.
Type	*Bauhinia divaricata* L.	*Barklya syringifolia* F. Muell.	*Gigasiphon humblotianum* (Baill.) Drake	*Lasiobema scandens* (L.) de Wit	*Lysiphyllum cunninghamii* (Benth.) de Wit	*Phanera coccinea* Lour.	*Piliostigma reticulatum* (DC.) Hochst.	*Schnella macrostachya* Raddi	*Tylosema fassoglense* (Schweinf.) Torre et Hillc.
Habit	Trees, shrubs (rarely semi-scandent), sometimes with intrastipular spines, rarely with thorns, without tendrils	Trees (unarmed, up to 18 m tall)	Mostly trees, occasionally large shrubs or climbers, without tendrils	Tendrilled lianas, scandent shrubs, without intrastipular spines or thorns	Trees, semi-scandent shrubs, tendrilled lianas, without intrastipular spines or thorns	Tendrilled lianas, scandent shrubs, rarely trees, without intrastipular spines or thorns	Trees, shrubs, without tendrils	Tendrilled lianas, scandent shrubs, without intrastipular spines or thorns	Trailing or climbing herbs, lianas, without intrastipular spines or thorns
Calyx	Spathaceous, splitting along one side or into 2 unequal lobes	Shortly lobed in the upper part, campanulate with obtuse teeth	Lobed, forming a very long hypanthium	Lobed or truncate	Lobed or split, broadly campanulate, ribbed, rusty-velvety	Lobed, forming 4 or 5 approximately equal lobes	Lobed in the upper part, dentate	Lobed or truncate, five-veined or inconspicuously veined	Lobed, forming a short hypanthium
Fertile stamen	1-10	10	10	3	10	3, rarely 2	10	10	2
Distribution	Pantropics	Australia	West and East Africa, Malesia	Asia	Australia, Southeast Asia	South and Southeast Asia	Africa, Asia, Australia	Neotropics	Africa
Species number	154	1	6	22	8	92	3	49	5

**Fig. 2:**
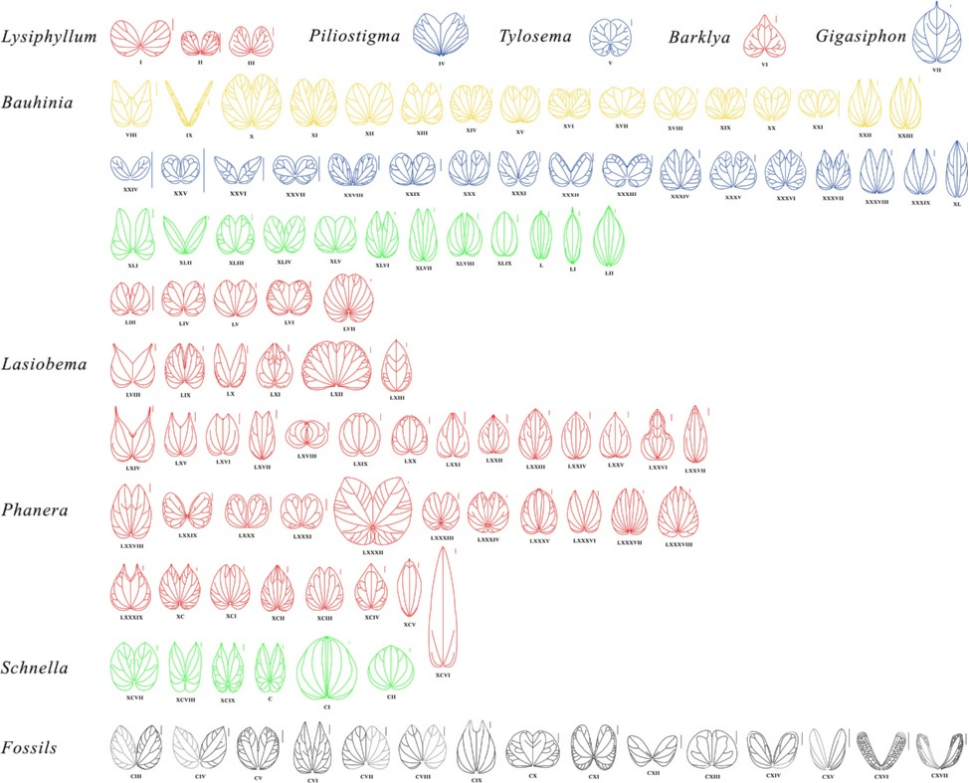
A diagram showing the leaf architecture of *Bauhinia* L. s.s. and its allies across the world. *Red*: Asian and Australasian species. *Blue*: African species. *Green*: American species. *Yellow*: Transoceanically distributed species. *Black*: fossil-species. (I-CXVII) Representative species (see Additional file 3). Scale bars = 1 cm

Phylogenetic relationships as well as divergence times of *Bauhinia* and its allies have been inferred by molecular analyses [12, 13] (Fig. 1). However, the scenarios of their diversification and migratory routes through time are little known due to the lack of a comprehensive study of the fossil record of *Bauhinia. Bauhinia* and its allies in the subtribe Bauhiniinae (Benth.) Walp. usually bear bilobate and bifoliolate leaves with pulvinate petiole and basal actinodromous or acrodromous venation [23–27] (Fig. 2I-CII; see Additional file 2), which are easily recognized in the fossil record (Fig. 2CIII-CXVII; see Additional file 2). *Bauhinia* and *Bauhinia*-like bilobate and bifoliolate leaf fossils have been reported from the Cenozoic of Asia, Africa, North America, and South America [28–44] (Table 2), providing the opportunity to evaluate the early evolution, diversification, and biogeographic history of the orchid trees from an historical perspective.

**Table 2:** The leaf fossil record of *Bauhinia* previously reported and used in this study

Fossil-species	Leaf size^a^	Illustration and notes	Locality	Geological age	Reference
*Bauhinia* sp.1	Ca. 2.2 × 2.0 cm	Poorly illustrated	Mahenge Site, Singida Town, Tanzania	Middle Eocene	[28]
*B. cheniae* Q. Wang et al.	Ca. 2.0–6.0 × 2.2–6.5 cm	Fig. 2CIII here	Ningming County, Guangxi Zhuang Autonomous Region, China	Possibly late Eocene or Oligocene	[29, 30]
*B. larsenii* D.X. Zhang et Y.F. Chen	Ca. 2.1–4.5 × 1.8–4.8 cm	Fig. 2CV here			
*B. ningmingensis* Q. Wang et al.	Ca. 4.0–5.3 × 4.0–5.2 cm	Fig. 2CIV here			
*Bauhcis moranii* Calvillo-Canadell et Cevallos-Ferriz	4.3–4.5 × 5.8–6.4 cm	Fig. 2CX; attributed to *Bauhinia* by some authors	Los Ahuehuetes, Tepexi de Rodríguez, Puebla, Mexico	Oligocene	[29–31]
*Bauhinia krishnanunnii* A.K. Mathur et al.	6.0 × 5.0 cm	Fig. 2CIX here	Dagshai Cantonment and Daghota, Kalka-Shimla Highway, Solan District, Himachal Pradesh, India	Early Miocene	[32]
*B. kachchhensis* R.N. Lakh. et Guleria	Ca. 6.2–8 cm wide, at least 3.5–5 cm long	Incompletely preserved	Khari Nadi Bed, near Goyela-Mokra; Thingdawl, Mizoram; Kachchh, India	Early–Middle Miocene	[33–35]
*Bauhinia* sp. 2	7.0 cm wide, at least 3.0 cm long	Incompletely preserved	Mae Sot, Changwat Tak, Thailand	Late Early Miocene–early Middle Miocene	[36]
*B. ungulatoides* sp. nov.	7.5–9.5 × 5.4–6.0 cm	Figs. 4 and 5 here	Lindai Village, Fotan Town, Zhangpu County, Zhangzhou City, Fujian Province, Southeast China	Middle Miocene	[37, this study]
*B. fotana* F.M.B. Jacques et al. emend.	Ca. 4.5–7.5 × 4.0–6.0 cm	Fig. 6 here			
*B. ramthiensis* Antal et N. Awasthi	Ca. 9.0 × 8.6 cm	Incompletely preserved	Right bank of upsteam of Ramthi River near Oodlabari, Darjeeling District, West Bengal, India	Middle Miocene	[38]
*B. siwalika* R.N. Lakh. et N. Awasthi	1.5–4.0 × 2.0–6.0 cm	Fig. 2CXIII here	Siwalik, Bhikhnathoree, West Champaran District, Bihar; Cherrapunji, West Khasi Hills District, Meghalaya, India	Middle Miocene–middle Pleistocene	[39]
*B. ecuadorensis* E.W. Berry	5.25 × 5.0 cm	Fig. 2CXI here	Loja Basin, Ecuador	Miocene	[40]
*B. wenshanensis* H.H. Meng et Z.K. Zhou	Ca. 6.0–7.0 × 3.0–4.0 cm	Fig. 2CVI here	Dashidong Town, Wenshan County, Southeast Yunnan Province, China	Late Miocene	[41]
*B. nepalensis* N. Awasthi et N. Prasad	4.5–7.5 × 5.6–11.4 cm	Fig. 2CVII here	Surai Khola beds, near Surai Khola bridge, Surai Khola, Nepal	Late Miocene–late Pleistocene	[42]
*B. waylandii* R.W. Chaney	2.5 × 2.5 cm	Fig. 2CXII here	Busano, Bugishu District, Eastern Province, Uganda	Pliocene	[43]
*Bauhinia* sp. 3	5.5 × 7.0 cm	Fig. 2CVIII here	Mahuadanr Valley, Palamu District, Bihar, India	Neogene	[44]

^a^As far as the bifoliolate-leafed species are concerned, each leaf is viewed twice as wide as one leaflet

In this article, we comprehensively investigate the leaf architecture of extant *Bauhinia* and its allies, describe two bilobate leafed fossil-species of *Bauhinia* from the middle Miocene Fotan Group of Fujian Province, southeastern China, and discuss their biogeographic implications.

## Methods

### Macrofossils

**Fig. 3:**
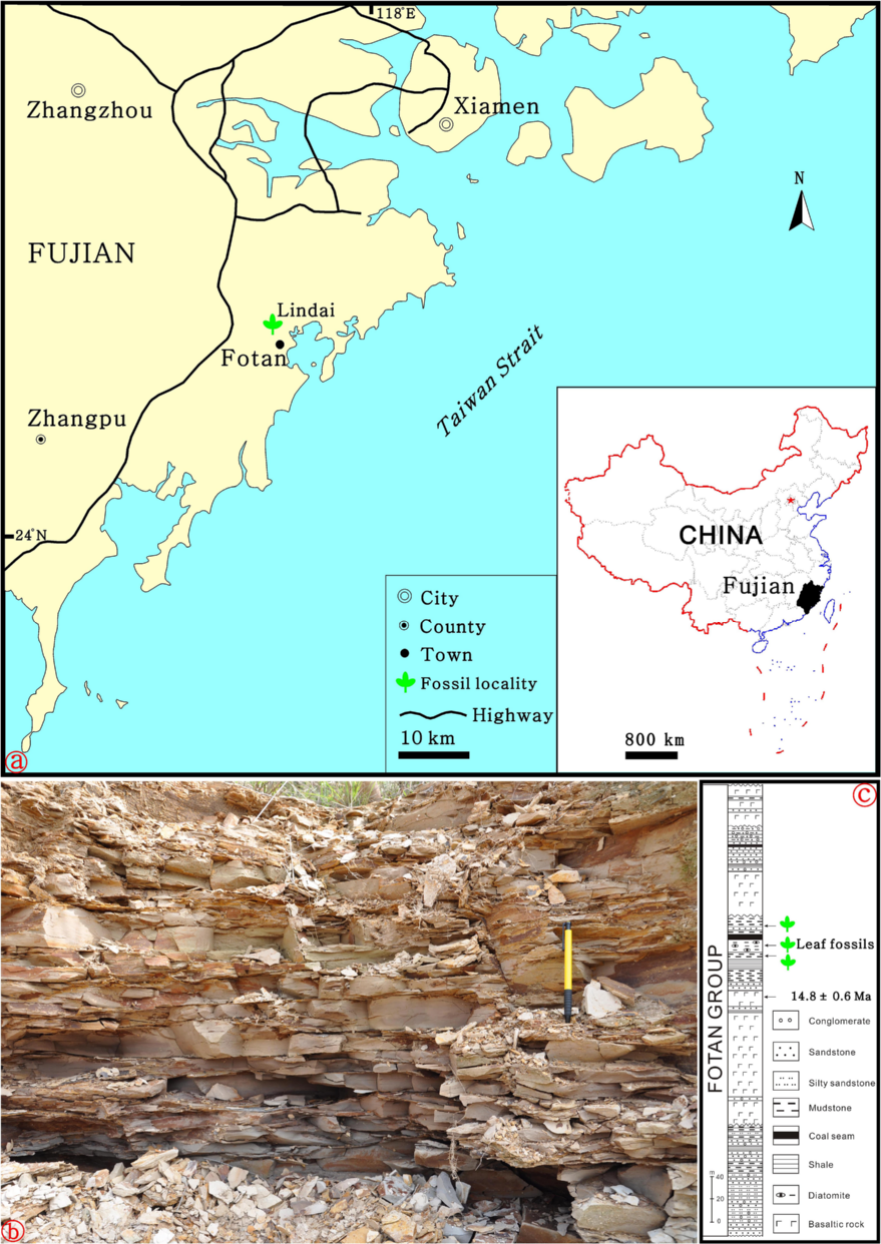
A diagram showing the fossil locality and stratigraphy. The maps and images are made and completed by the present authors. **a** A map indicating the fossil location. **b** The sampling site. **c** A stratigraphical column of the Fotan Group

The fossil leaves investigated in this paper were collected from the Fotan Group at Lindai Village (lat. 24°12′N, long.117°53′E) of Zhangpu County, Fujian Province, southeastern China (Fig. 3). Paleobotanical fieldwork was done in non-National Nature Reserves and non-private areas with the permission of the local government. The stratigraphy of the Fotan Group has previously been discussed in detail [45, 46]. Generally, it consists of basaltic rocks, arenaceous conglomerate rocks, sandstone and mudstone interbedded with lignite and diatomite. The outcrop at Lindai Village (i.e., sampling site) is composed of an upper layer of light-brown diatomite and an underlying layer of blue-gray mudstone (Fig. 3b, c). Both layers yield abundant plant fossils dominated by angiosperm leaves, but fruits are also present [47, 48]. The fossils from the diatomite layer are commonly preserved as impressions with exquisite venation while those from the mudstone layer are often preserved as compressions with cuticle. The geological age of the Fotan flora in Zhangpu is considered the Langhian Stage of middle Miocene [49] on the basis of an Argon-Argon (^40^Ar/^39^Ar) radiometric dating (14.8 ± 0.6 Ma) of the basaltic rocks underlying the fossil-bearing layers (Fig. 3c).

The studied fossil leaves were examined and photographed using digital cameras (Panasonic DMC-FZ30 and Nikon D90). The cuticles were macerated using Schulze’s solution followed by diluted Ammonia (for a detailed procedure see [50]), and then mounted on slides, examined and photographed on a Zeiss AxioScope A1 microscope. Both the figured macrofossil specimens and cuticle slides (prefixed “PB”) are deposited at Nanjing Institute of Geology and Palaeontology, Chinese Academy of Sciences, Nanjing, P.R. China.

In addition, we examined the previously reported *Bauhinia* leaf fossils from the Oligocene Ningming Formation of Guangxi, South China [29, 30] and the Neogene of India and Nepal [38, 39, 42, 44]. The specimens are housed at Natural History Museum of Guangxi, Nanning (NHMG) and Birbal Sahni Institute of Palaeobotany, Lucknow (BSIP) (see Additional file 3).

### Herbaria

The exsiccatae used in this study are from the following Herbaria: Harvard University, Cambridge (A), The Natural History Museum, London (BM), National Botanic Garden of Belgium, Meise (BR), Queensland Herbarium, Brisbane (BRI), Royal Botanic Garden Edinburgh, Edinburgh (E), Field Museum of Natural History, Chicago (F), Centro Studi Erbario Tropicale Università degli Studi di Firenze, Firenze (FT), Conservatoire et Jardin Botaniques de la Ville de Genève, Genève (G), Royal Botanic Gardens, Kew (K), National Herbarium Nederland, Leiden University Branch, Leiden (L), Botanische Staatssammlung München, München (M), Real Jardín Botánico, Madrid (MA), National Herbarium of Victoria, Melbourne (MEL), Missouri Botanical Garden, Missouri (MO), The New York Botanical Garden, Bronx (NY), Muséum National d’Histoire Naturelle, Paris (P), The Chinese National Herbarium, Beijing (PE), the Swedish Museum of Natural History, Stockholm (S), Trinity College, Dublin (TCD), Smithsonian Institution, Washington (US), and Wageningen University, Wageningen (WAG) (see Additional file 3).

### Online databases

(1) *ILDIS* (International Legume Database & Information Service) [51]. The species and distribution of *Bauhinia* and its allies have been compiled by *ILDIS*, with special reference to some recently published taxonomic articles (Table 1; see Additional file 1). (2) *eFloras.org* [52]. Morphological descriptions and illustrations of *Bauhinia* and its allies concerned here were checked. (3) *Chinese Virtual Herbarium (CVH)* [53]. Online images of herbarium specimens of *Bauhinia* and its allies were browsed. (4) *Index Herbarium* [54]. The standardized Herbarium codes were adopted. (5) *The International Plant Names Index* [55]. The standardized abbreviations for authors of plant-names and journal titles in References were consulted and adopted in this paper.

### Terminology

The gross morphology, venation, and cuticle of modern and fossil leaves were described on the basis of the standard terminology [56, 57]. The morphological interpretation and terms specifically for leaves of the Cercideae follow the literature [24, 27, 30]. Time calibrations and geological terms referred to the latest International Chronostratigraphic Chart [58].

### Figures

**Fig. 4:**
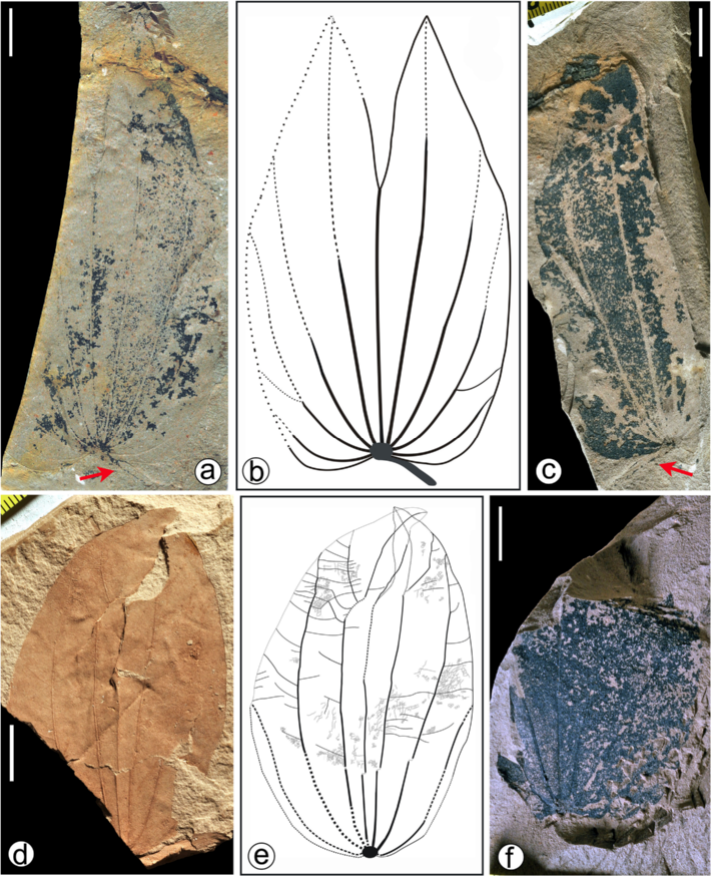
Leaf morphology of *Bauhinia ungulatoides* sp. nov. from the middle Miocene of Fujian Province, southeastern China. **a**-**c** Holotype, PB21584 a, b and its line drawing, indicating extremely ascending primary veins (1°) and acute apices. Red arrows refer to a partially preserved petiole. **d**-**e** PB21585, showing extremely ascending 1°, but slightly deformed due to preservational crushing. **f** PB21586, indicating a partially preserved leaf with a base and similar 1° to those in (**a**-**c**). Scale bars = 1 cm

**Fig. 5:**
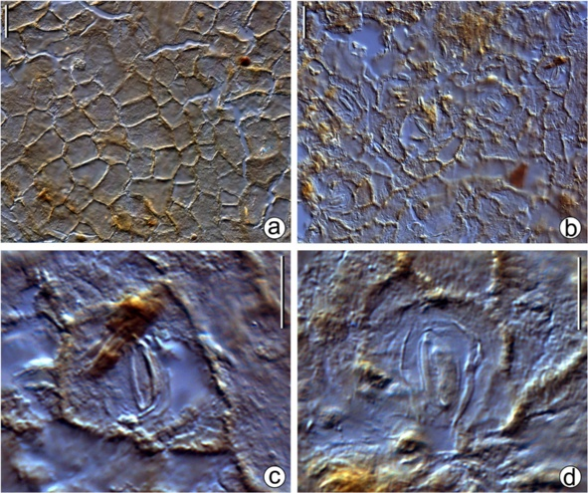
Leaf micromorphology of *Bauhinia ungulatoides* sp. nov. from the middle Miocene of Fujian Province, southeastern China. The cuticles from the holotype, PB21584 c, d. **a** Adaxial cuticle showing the morphology of epidermal cells. **b** Abaxial cuticle showing the orientation of stomata. Scale bars = 20 μm. **c**-**d** showing an enlarged paracytic stomatal complex from (**b**). Scale bars = 10 μm

**Fig. 6:**
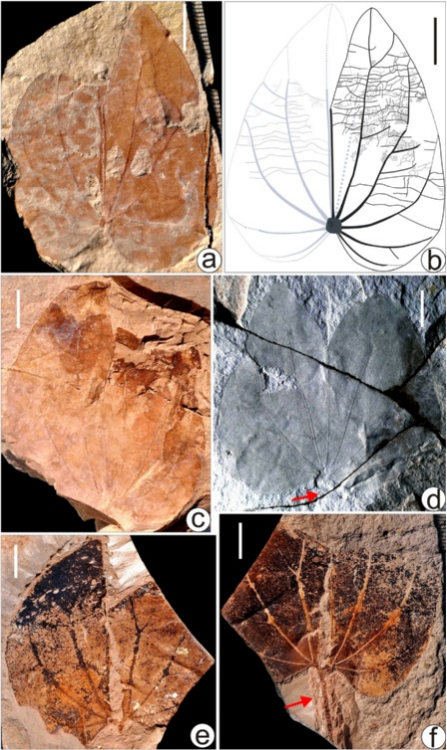
Leaf morphology of *Bauhinia fotana* from the middle Miocene of Fujian Province, southeastern China. **a**-**b** Epitype, PB21579 and its line-drawing, showing the detailed leaf architecture. Note that this leaf was torn along the midvein and then became partially overlapped of two lobes after a twist during the process of fossilization. **c** PB21580, showing basal actinodromous primary veins (1°). Note that the apical parts of the leaf were deformed. **d** Holotype, PB21577, indicating a relatively complete leaf with stout, basal actinodromous 1°. **e**-**f** PB21581a, b, showing the part and counterpart specimens of a leaf. Note that this leaf was torn near the midvein, and the right lobe was partially folded. Red arrows in (**d**, **f**) refer to a partially preserved petiole. Scale bars = 1 cm

A simplified diagram (Fig. 1) showing the phylogenetic relationships and divergence times of *Bauhinia* and its allies was redrawn from literature [12, 13]. The diagram of the locality and strata (Fig. 3) as well as line-drawings of both modern and fossil leaves (Figs. 2, 4 and 6) were drawn using CorelDRAW 12.0 program, and photographs of the sampling site and specimens were combined into figures using CorelDRAW 12.0 program (Figs. 2, 3, 4, 5 and 6).

### Leaf morphological analyses

Leaf morphological variables are measured and calculated using CorelDRAW 12.0 on the basis of leaf images (Fig. 2) from illustrated species, including the length-to-width/2 ratio, dissection index, and sinus (see Additional file 4). The dissection index (DI) is the ratio of an outline’s perimeter to the square root of its area [59–61], i.e., DI = Perimeter/[2sqrt (π × Area)], which is a standardized metric to determine shape complexity, especially regarding the complex degree of bilobate leaves studied here.

## Results

### Leaf morphology of *Bauhinia* and its allies

The leaf architecture of 100 representatives of the Bauhiniinae including 15 fossil species is illustrated here (Fig. 2I-CXVII; see Additional file 2 for each species name), accounting for ca. 1/3 of the species in the subtribe. Leaves of Bauhiniinae (Table 1; see Additional file 1) are generally characterized by unifoliolate, bilobate and bifoliolate types, and the bilobate type is the commonest (Fig. 2). By observing and analyzing 10 characters, i.e., length-to-width/2 ratio, dissection index (Perimeter/[2sqrt (π × Area)]), apex, base, lobation, sinus, texture, primary veins (1̊), secondary veins (2̊), and epidermal anatomy [62–69], we compared the leaf architecture and morphological complexity of *Bauhinia* and its allies (Table 3; see Additional file 4).

**Table 3:** Leaf morphological comparisons between *Bauhinia* and its allies (Fig. 2; see Additional file 4)

Taxon names	*Bauhinia* L. s.s. outside America	*Bauhinia* L. s.s. in America	*Bauhinia ungulatoides* Y.X. Lin et al.	*Bauhinia fotana* F.M.B. Jacques et al.	*Barklya* F. Muell.	*Gigasiphon* Drake	*Lasiobema* (Korth.) Miq.	*Lysiphyllum* (Benth.) de Wit	*Phanera* Lour.	*Piliostigma* Hochst.	*Schnella* Raddi	*Tylosema* (Schweinf.) Torre et Hillc.
Length/(Width/2)	2.191	3.306	3.439	2.329	2.101	2.572	2.798	1.422	2.750	1.702	2.612	1.761
Dissection index (Perimeter/[2sqrt (π × Area)])	1.350	1.320	1.388	1.322	1.183	1.181	1.232	1.447	1.313	1.121	1.490	1.198
Apex	Obtuse, acuminate or acute	Acuminate or acute, rarely obtuse	Acute	Acute or slightly obtuse	Acuminate	Acuminate, with a drip tip	Acuminate or acute, rarely obtuse	Obtuse or rounded	Obtuse, acuminate or acute	Obtuse	Acuminate or acute	Rounded
Base	Cuneate, truncate, or Slightly to deeply cordate	Cuneate, truncate or slightly cordate, rarely deeply cordate	Slightly or shallowly cordate	Moderately to deeply cordate	Deeply cordate	Slightly cordate	Slightly to deeply cordate or cuneate, rarely truncate	Slightly to deeply cordate	Slightly to deeply cordate, raely cuneate	Cuneate	Slightly to deeply cordate	Moderately cordate
Lobation	Bilobate or bifoliolate, rarely unifoliolate	Bilobate or unifoliolate, rarely bifoliolate	Bilobate	Bilobate	Unifoliolate	Unifoliolate	Bilobate or unifoliolate	Bifoliolate	Bilobate, bifoliolate or unifoliolate	Bilobate	Bilobate, rarely bifoliolate	Unifoliolate
Texture	Chartaceous to coriaceous	Mainly coriaceous	Coriaceous	Chartaceous	Chartaceous	Coriaceous	Mainly chartaceous	Chartaceous	Chartaceous to coriaceous	Mainly coriaceous	Mainly coriaceous	Chartaceous
Sinus	No or < 120°	No or < 80°	20°–30°	30°–45°	No	No	No or < 130°	15°–30°	No or < 75°	80°–90°	No or < 25°	70°–80°
1° veins	Actinodromous or acrodromous	Actinodromous or acrodromous	Actinodromous	Actinodromous	Actinodromous	Actinodromous	Actinodromous or acrodromous	Actinodromous	Actinodromous or acrodromous	Actinodromous	Actinodromous or acrodromous	Actinodromous
	5–13 in number Not branched to frequently branched	5–9 Not branched or less branched, frequently branched rarely	7–9 Less branched	9–11 Branched	7 Frequently branched	7 Branched	5–9 Not branched to frequently branched	8–10 Frequently branched	5–13 Not branched to frequently branched	11 Frequently branched	7–11 Not branched to frequently branched	9 Frequently branched
2° veins	Craspedodromous, eucamptodromous or brochidodromous	Craspedodromous, eucamptodromous, rarely brochidodromous	Craspedodromous	Eucamptodromous	Cladodromous	Eucamptodromous	Brochidodromous or eucamptodromous	Craspedodromous or eucamptodromous	Brochidodromous craspedodromous, 1 or eucamptodromous	Simple brochidodromous	Craspedodromous, eucamptodromous or brochidodromous	Cladodromous
Epidermal anatomy ([7, 17–19, 21, 22, 62–69], this paper)	Epidermal walls straight, wavy or sinuate; stomata paracytic, anomocytic, anisocytic or tetracytic; trichomes multicellular, uniseriate, unicellular or no; glands present or not	Epidermal walls straight or wavy; stomata anomocytic or paracytic, trichomes uniseriate or multiseriate, glandular; glands present or not	Epidermal walls straight or slightly curved; stomata paracytic; no trichome; no gland	Not preserved	Trichomes sometimes sparse, caducous; minute intrastipular trichomes	Subglabrous or almost glabrous, with minute trichomes on the veins	Epidermal walls straight; stomata tetracytic; no trichome	Epidermal walls wavy or sinuate; stomata tetracytic; no trichome	Epidermal walls wavy or sinuate; stomata anisocytic, tetracytic, anomocytic or paracytic; trichomes on both surfaces; no gland	Epidermal walls straight; stomata anomocytic or anisocytic; trichomes multicellular, unicellular, uniseriate and hooked; no gland	Epidermal walls undulate or sinuate; trichomes glandular, multicellular, uniseriate; no gland	Trichomes linear, canaliculate, with a ring and conical base or not; no gland

### Systematics

Family Leguminosae Juss.

Subfamily Caesalpinioideae DC.

Tribe Cercideae Bronn

Subtribe Bauhiniinae (Benth.) Walp.

Genus *Bauhinia* L. s.s.

Type *Bauhinia divaricata* L.

### Fossil species

Two fossil-species of *Bauhinia* are described as follows. All the voucher specimens were collected from the same locality and stratigraphy and are deposited at the same institute.

### Type locality

Lindai Village, Zhangpu County, Zhangzhou City, Fujian Province, P. R. China (Fig. 3a).

### Stratigraphic horizon and age

The middle part of the Fotan Group, Langhian Stage (middle Miocene) (Fig. 3b, c).

### Repository

Nanjing Institute of Geology and Palaeontology, Chinese Academy of Sciences, Nanjing, P.R. China.

***Bauhinia ungulatoides*** Y.X.Lin, W.O.Wong, G.L.Shi, S.Shen et Z.Y.Li, sp. nov. (Figs. 4 and 5).

### Etymology

The specific epithet is derived from the Latin “*ungulatus*” (hoof-shaped) suffixed by “*oides*” (resembling), implying the striking similarities between leaves of studied fossils and extant *Bauhinia ungulata* L. (Fig. 2XXIII).

### Holotype

PB21584a, b, c, d (Fig. 4a, c; Fig. 5a, b) (designated here. A gathering with part and counterpart specimens, including slides of leaf cuticles).

### Paratypes

PB21585 (Fig. 4d), PB21586 (Fig. 4f) (designated here).

### Diagnosis

Lamina ovate-elliptical or elliptical in outline, shallowly to moderately bilobate, with pulvinate petiole and untoothed margin. Laminar base slightly or shallowly cordate, lobe apex acute. Primary venation basal actinodromous with 7–9 basal veins. Midvein terminated at the bottom of a narrow sinus. Lateral primaries straight or curved, and the innermost pairs reaching the lobe apex and outer pairs approaching to the laminar margin. Secondary veins craspedodromous. Intersecondary veins present. Tertiary veins opposite and alternate percurrent. Quaternary veins forming irregular polygons. Areolation well developed. Freely ending veinlets unbranched. Marginal ultimate veins absent; leaves hypostomatic. Epidermal cells on both surfaces, similarly quadrangular or pentagonal, with straight or slightly curved anticlinal walls. Stomatal complexes paracytic, randomly oriented.

### Description

The leaf attachment is petiolate. Petioles are partially preserved, at least 1.5 cm long, with a tiny, semicircular upper pulvinus impression connecting the laminar base (Fig. 4a-c). Laminae are bilobate, ovate-elliptical or elliptical in outline (Fig. 4a-e), ca. 7.5–9.5 cm long and 5.4–6.0 cm wide. The apex is bifid to ca. 1/3–2/5 of laminar length, with a reflex apex angle at ca. 20°–30° (Fig. 4a, b, d, e). Two lobes are symmetrical or slightly asymmetrical. Lobe apices are acute. Laminar bases are slightly or shallowly cordate (Fig. 4a-f). The margin is entire. The texture appears coriaceous. Primary venation is basal actinodromous with 7–9 basal veins. The midvein is straight, moderate in thickness, terminated at the bottom of the sinus. Lateral primaries are straight or curved, extremely ascending and rarely branched, and the innermost pairs reaching the lobe apex and outer pairs approaching to the laminar margin (Fig. 4a-f). Secondary veins are less prominent, craspedodromous, diverging at ca. 40°–80° from the innermost and outmost lateral primaries and approaching to the laminar margin (Fig. 4a, b, d, e). Intersecondary veins are approximately parallel to neighboring secondary veins, radiating out about 1/3–1/2 of distance from the primaries to laminar margin. Tertiary veins are opposite and alternate percurrent, slightly curved to sinuous, connecting the neighboring primary veins or between the primary veins and the secondary veins (or the margin). Quaternary veins are linked with other higher veins, forming irregular polygons (Fig. 4d, e). The areolation is well developed. Freely ending veinlets are unbranched. Marginal ultimate veins are absent.

Leaves are hypostomatic. The adaxial cuticle consists of isodiametric to slightly elongated epidermal cells. These cells are usually quadrangular or pentagonal, rarely hexagonal or heptagonal, with straight to slightly curved anticlinal walls and smooth periclinal walls (Fig. 5a). Epidermal cells in abaxial cuticles are similar in form and size to those in adaxial cuticles. Stomata are evenly distributed in the non-venous region of abaxial cuticles and randomly oriented (Fig. 5b). Stomatal complexes are paracytic, commonly asymmetrical, ovate, elliptical to oblong in outline. Subsidiary cells are crescent or irregularly shaped. Guard cells are not sunken (Fig. 5c, d). Trichomes or trichome bases are not observed.

### Comparisons

The bilobate leaves described here are obviously different from five small genera, i.e., *Lysiphyllum, Piliostigma, Tylosema, Barklya* and *Gigasiphon* (Fig. 2I-VII; Table 3). Leaves of extant *Tylosema*, *Barklya* and *Gigasiphon* are unlobed or only very slightly bilobate; *Lysiphyllum* are bifoliolate. *Piliostigma* has bilobate leaves like our fossil leaves, but differs in having more basal veins (11) and bigger reflex apex angle (80°–90°). Following comparison with extant *Bauhinia*, *Lasiobema*, *Phanera* and *Schnella* (Fig. 2VIII-CII; Table 3), the present leaf fossils are attribute to *Bauhinia* in the light of character combinations such as very similar leaf architecture and dissection index. Leaves of *Schnella* have cordate, even auriculate bases (Fig. 2XCVII-C). In *Phanera*, leaves are usually broader than [lower in L/(W/2) ratio] than *B. ungulatoides* (Table 3; see Additional file 4), and the widest part is near the base (Fig. 2LXXXVII-XCIII). *Bauhinia ungulatoides* are similar to *Phanera coccinea* Lour. (Fig. 2LXXVIII), but are more shallowly bilobate. It can also be easily distinguished from the bilobate leaves of *Lasiobema* by reflex apex angles at ca. 20°–30°. The reflex apex angles of leaves in *Lasiobema* are either very wide (>40°) (Fig. 2LVIII, LX, LXIV-LXVII) or extremely narrow (<10°) sinus (Fig. 2LIX, LXI, LXXI, LXXII) (see Additional file 4). *Bauhinia ungulatoides* is characterized by shallowly cordate bases and acute apices on each lobe. It is more or less distinguishable from the bilobate leaves of *Bauhinia*’s allies in Bauhiniinae (Table 3). Although it is possible that these similarities are result of convergent evolution it is worth noting that *Bauhinia ungulatoides* is most similar to two South American species, i.e., *B. ungulata* (Fig. 2XXIII) and *B. forficata* Link [26, 63] (Fig. 2XXII), among the investigated extant species. They all bear extremely ascending and rarely branched lateral primary veins. However, since the reproductive organs of *B. ungulatoides* are unknown, it is more appropriate to assign it to a new fossil-species rather than to any extant species. Regarding fossil-species (Table 2), *B. ungulatoides* is similar to *B. wenshanensis* H.H. Meng et Z.K. Zhou from the late Miocene of Yunnan, southwestern China [41] (Fig. 2CVI), but the latter bears more secondary veins.

***Bauhinia fotana*** F.M.B.Jacques, G.L.Shi et Z.K.Zhou emend. Y.X.Lin, W.O.Wong, G.L.Shi, S.Shen et Z.Y.Li (Fig. 6).

*Bauhinia fotana* F.M.B. Jacques, G.L. Shi et Z.K. Zhou in Jacques et al., *Rev. Palaeobot. Palynol*. 216: 78, Fig. 3a, pl. 1, Figs. 1, 2, 2015.

### Holotype

PB21577 (Fig. 6d herein) (first designated and illustrated by Jacques et al. [37]).

### Epitype

PB21579 (Fig. 6a, b) (An epitype is selected and designated here under Article 9.8 of the *ICN* (Melbourne Code) [70] to display the detailed leaf architecture that the holotype lacks).

**Paratype** (first designated and illustrated by Jacques et al. [37]).

PB21578.

### Other specimens examined here

PB21580 (Fig. 6c), PB21581a, b (Fig. 6e, f), PB21582, and PB21583.

### Emended description

The leaf attachment is petiolate. Petioles are partially preserved, at least ca. 1/2 of laminar length, with a tiny, semicircular upper pulvinus impression connecting the laminar base (Fig. 6a, b, e, f). Laminae are bilobate, broadly ovate to suborbicular in outline (Fig. 6a-f), ca. 4.5–7.5 cm long and 4.0–6.0 cm wide. The apex is bifid to ca. 1/3 of laminar length, with a reflex apex angle at ca. 30°–45° (Fig. 6b, d). Two lobes are symmetrical or slightly asymmetrical. Lobe apices are acute or slightly obtuse. Laminar bases are moderately to deeply cordate (Fig. 6a-f). The margin is entire. The texture appears chartaceous. Primary venation is basal actinodromous with 9–11 basal veins. The midvein is stout in thickness, terminated at the bottom of the sinus. The outmost pairs are weaker than the midvein and inner pairs. Lateral primaries are curved, branched or unbranched, and the innermost pairs reach the lobe apex and outer pairs approaching to the laminar margin (Fig. 6a-f). Secondary veins are eucamptodromous, diverging at ca. 45°–80° from the innermost and outmost lateral primary veins and approaching to the laminar margin (Fig. 6a-b). Tertiary veins are opposite and alternate percurrent, mostly sinuous and convex, rarely straight, connecting the neighboring primary veins or between the primary veins and the secondary veins (or the margin), as well as forming agrophic veins to the margin at the laminar base (Fig. 6a-b). Quaternary veins are linked with other higher veins, forming irregular polygons (Fig. 6b). The areolation is well developed. Freely ending veinlets are unbranched. Marginal ultimate veins are absent. Cuticles are unavailable.

### Comparisons

*Bauhinia fotana* was originally described on the basis of two fossil leaves [37], but the initial description is very simple without characters of high order veins known. Here, we emended it based upon its types and newly collected specimens from the same locality. Its leaves are broader [smaller in L/(W/2) ratio] than *Schnella* (Fig. 2XCVIII-C) and most *Phanera* species (Fig. 2LXXVIII, LXXXVI-LXXXVIII) (Table 3, see Additional file 4). *Bauhinia fotana* is somewhat similar to *Phanera ornata* (Kurz) Thoth. (Fig. 2XC), but bears more deeply bilobate leaves with much less secondary veins. It is different from *Lasiobema*, which usually has an obvious caudate apex (Fig. 2LVIII, LXIV, LXV). Except for the size (4.5–7.5 × 4.0–6.0 cm), *B. fotana* shows great similarities with the extant *B. acuminata* L. (9–12 × 8–12.5 cm) (Fig. 2XIII) in bearing broadly ovate or suborbicular bilobate leaves with an acute apex, a moderately to deeply cordate base as well as similar venation. Among the fossil species (Table 2), *B. fotana* closely resembles *Bauhinia* sp. 3 (Fig. 2CVIII) from the Neogene of India [44], but the latter bears weak primary veins and larger L/(W/2) ratio of leaves.

## Discussions

The pantropical genus *Bauhinia* and its allies have similarly bilobate, bifoliolate, or unifoliolate leaves. They along with the northern temperate to subtropical genus *Cercis* L. constitute the tribe Cercideae as sister to the remaining legumes in the molecular phylogenetic trees [1–5, 10–13] (Fig. 1). Recently, strictly east-to-west vicariances for the biogeographic evolution of *Cercis* and *Bauhinia* have been suggested through molecular analyses [41, 71]. The earliest diverging clades in the Bauhiniinae were inferred to make their debut most possibly in Asia during the middle Paleocene (ca. 62.7 Ma) [41]. The fossil record of *Cercis* and *Bauhinia* can provide key points of reference for deciphering the early evolution and biogeographic history of the Cercideae.

Bilobate fossil leaves that are attributed to or closely compared with *Bauhinia* are also reported from the late Eocene of Vietnam [72], the late Eocene-early Miocene of Brazil [73], and the latest Oligocene-mid-late Miocene of Australia [74]. These records, however, have been either rejected or questioned due to lack of evidence for the pulvinus and/or basal actinodromous or acrodromous venation [30]. Paleobotanical evidence indicates that *Cercis* [75] and *Bauhinia* [30] (Table 2) had first appeared in the Eocene to Oligocene of mid-low latitudes in the Northern Hemisphere. This may more or less support a tropical Tethys Seaway (Laurasian) origin [4, 5, 13] or an “Out of Tropical Asia” dispersal [25, 41] of the Cercideae and the Leguminosae as previously hypothesized. In contrast, the West Gondwana hypothesis or “Out of Africa” hypothesis for the origin of legumes [76–78] has been recently rejected by biome supertree and molecular analyses [4, 5]. In this article, bilobate leafed fossil-species, i.e., *B. ungulatoides* and *B. fotana*, from the middle Miocene of Fujian, southeastern China provide some new insights into the biogeography of *Bauhinia* and its allies*.*

### Floristic exchanges between East Asia and South Asia

Major collision of India with Asia in the early Cenozoic enlarged the land-area linked to Eurasia, and subsequent connection with Australasia during the Neogene led to more connections between Eurasia and Oceania [79–82], which have greatly facilitated the floristic exchanges between East Asia, South Asia, Southeast Asia and Oceania. Recent paleobotanical studies have suggested that the Sino-Indian floristic affinities have begun to be established between the tropical flora of India and (sub) tropical floras of southwestern and southeastern China during the Miocene [37, 83–85]. Our present study on the Miocene *Bauhinia* further supports this viewpoint.

Bilobate leaves of *Bauhinia* from the Miocene of southeastern China show considerable similarities with the congeneric fossil-species [41, 44] from the Neogene of southwestern China and India, implying that the expansion of *Bauhinia* from (sub) tropical East Asia to tropical South Asia might have taken place since the Miocene with the northward drift and collision of southern landmasses into Eurasia, as well as the closure of the eastern Tethys Seaway [86]. The fossils presented here further support the previous viewpoint [30] that the tropical zone [87] of South China may represent one of the centers for early diversification of *Bauhinia*. The bilobate and bifoliolate leaves from the Oligocene (or possibly late Eocene) Ningming Formation of Guangxi, South China [30] are the earliest, well-documented, reliable fossils of *Bauhinia*.

### Floristic exchanges between Eurasia and Africa

Africa has been connected with Europe by the collision between the Afro-Arabian and Eurasian plates since the late Late Cretaceous [88], which facilitated floristic exchange such as the pantropical palms (Arecaceae Schultz Sch.) [78, 89]. *Bauhinia* and *Bauhinia*-like fossils previously reported from the early Paleocene to Miocene of Europe [90–95] have been either rejected or transferred to other groups [30]. Instead, some other bilobate leaf fossils that had been described as *Cassia* L. and *Mimosa* L. from the Oligocene of Germany and France [96–100] (Fig. 2CXIV-CXVII) are far more likely to represent *Bauhinia. Cassia rottensis* Weyland, *Mimosa weberi* Schimp., *M. deperdita* Saporta, and *M. ayamadi* Marion closely resemble the extant *Bauhinia* in their basal actinodromous or acrodromous venations. Specifically, *C. rottensis* and *M. weberi* from Germany bear great similarities with the extant African species *Bauhinia morondavensis* Du Puy et R. Rabev. (Fig. 2XXVIII), *B. natalensis* Hook. (Fig. 2XXX), and *B. kalantha* Harms. (Fig. 2XXXI).

Under such circumstances, *Bauhinia* and *Bauhinia*-like bilobate leaf fossils from the Oligocene of Germany (Fig. 2CXIV, CXV) as well as from the middle Eocene of Tanzania [28] imply that the Bauhiniinae might have begun to exchange between Europe and Africa across the western Tethys Seaway. *Bauhinia* might have become depauperate and finally extinct in Europe after the Oligocene with the uplift of the Himalayan-Tibetan plateau [101], the desertification in the Asian interior [102], the establishment of the Asian monsoon system [103], and the desiccation of the Mediterranean Sea [104].

### Migration from Eurasia to America via the North Atlantic Land Bridge

In America *Bauhinia* and *Bauhinia*-like bilobate leaves (Fig. 2CX, CXI) are only known from the Oligocene of Mexico [31] and the Miocene of Ecuador [40]. The *Bauhina* leaf from late Eocene-early Miocene of Brazil is questionable since it lacks pulvinus and the primary venation is not distinctly basal actinodromous [30]. Brazil that occupies highly diversified *Bauhinia* species today has been suggested as the center for origin of orchid trees [73]. However, recent molecular phylogenetic study resolved Asian species as the basalmost lineage in the genus *Bauhinia* whereas the neotropical species diverged during the middle Miocene [41]. The relatively extensive fossil record of *Bauhinia* from the late Paleogene – early Neogene of South China also supports that South China is one of the centers for early diversification of the genus.

Given that the Bauhiniinae originated in the Paleogene of low-latitudes along the eastern Tethys Seaway as we hypothesize here, it is most likely that *Bauhinia* and its allies migrated into North and Central America from Europe via the North Atlantic Land Bridge (NALB) [105–108]. During the early Paleogene, with the epicontinental seaways around North America and Eurasia receding, barriers between these two continents were reduced, allowing floristic exchanges of thermophilous plants to develop into a more uniform and continuous Boreotropical flora [78, 105, 106, 109]. The NALB lay at lower latitude in the Paleogene-early Neogene than the Bering Land Bridge (BLB), and it may have been more favorable for tropical, subtropical or even temperate plants to migrate [107–110]. So far, *Bauhinia* fossils are unknown in mid-high latitudes from East Asia and North America, supporting that the BLB, situated at higher latitudes, seems not to have witnessed the migration of *Bauhinia*. Hence, the NALB may have been the most feasible route for migration of *Bauhinia* from Eurasia to North America since the late Paleogene. An alternative migration route from Eurasia to North America for (sub) tropical lineages that have recently been suggested for *Smilax* Havanensis group [111] might also apply to the presumable trans-Atlantic dispersal of *Bauhinia*. These authors suggested trans-Atlantic crossings at lower latitudes via “floating islands” as has also been suggested for numerous angiosperm lineages [112] and for animals (e.g., platyrrhine monkeys) [113].

It is of great interest that bilobate leaves of *Bauhinia* presented here from the middle Miocene of southeastern China exhibit great similarities with some extant *Bauhinia* species (Fig. 2XXII, XXIII) from South America, implying extensive dispersals of *Bauhinia* populations from Eurasia to America in or by the Miocene, during which the Isthmus of Panama was formed, facilitating the Great American Biotic Interchange [114, 115]. This inference is also consistent with the result based on molecular phylogenetic study [41], which suggests that South American *Bauhinia* diverged during the middle Miocene.

## Conclusions

Bilobate leaf fossils, i.e., *B. ungulatoides* and *B. fotana* presented here, from the middle Miocene of southeastern China are consistent with the viewpoint that the tropical zone of South China is one of the centres for early diversification of *Bauhinia*, and their great similarities to some species from South Asia and South America imply that *Bauhinia* might have undergone extensive dispersals and diversification during the Miocene.

The reliable fossil record, extant species diversity, as well as molecular phylogenetic analyses suggest that the Bauhiniinae might have originated in the Paleogene of low-latitudes along the eastern Tethys Seaway. They dispersed southwards into Africa, migrated from Eurasia to North America via the North Atlantic Land Bridge or floating islands in southern North Atlantic during the Oligocene. Then they spread into South America via the Isthmus of Panama since the Miocene onward, and underwent regional extinctions in the Boreotropics of mid-high-latitudes by the Neogene climatic cooling, so *Bauhinia* presently exhibits a pantropical intercontinental disjunct distribution.

## Additional files

Additional file 1:
**The species and distribution of** ***Bauhinia*** **and its allies.** (PDF 266 kb) Additional file 2:
**The list of representative species in Fig.** 2 **.** (PDF 141 kb) Additional file 3:
**Information on voucher specimens used in this study.** (DOC 177 kb) Additional file 4:
**Morphological analyses of leaves from illustrated species in** ***Bauhinia*** **and its allies.** (PDF 158 kb)
