# Supplementary material for: Bilobate leaves of Bauhinia (Leguminosae, Caesalpinioideae, Cercideae) from the middle Miocene of Fujian Province, southeastern China and their biogeographic implications
Source: BMC Evol Biol. 2015 Nov 16;15:252. doi: 10.1186/s12862-015-0540-9 (PMC4647482; doi:10.1186/s12862-015-0540-9)
Supplement: Additional file 1: — The species and distribution of Bauhinia and its allies. (PDF 266 kb) [file 12862_2015_540_MOESM1_ESM.pdf]

Additional file 1: The species and distribution of *Bauhinia* L. s.s. and its allies (Tables 1-9).

Table 1 The species and distribution of *Bauhinia.* L. s. s.

**Accepted names (154 in number)**	**Distribution**
B. acreana Harms	Bolivia, Brazil, Peru
B. aculeata L.<br>(non *B. aculeata* Vell. = *B. forficata* Link)	India, Caribbean-TRP, El Salvador, Panama, Argentina, Bolivia, Ecuador, Peru, Venezuela
B. acuminata L.	Sierra Leone, Zaire, Bangladesh, Brunei, Cambodia, China (Guangdong, Guangxi, Yunnan, Taiwan), India (Arunachal Pradesh, Assam, Bihar, Goa, Gujarat, Karnataka, Kerala, Madhaya Pradesh, Maharashtra, Manipur, Meghalaya, Nagaland, Orissa, Pondicherry, Punjab, Rajasthan, Tamil Nadu, Tripura, Uttar Pradesh, West Bengal), Indonesia-ISO, Java, Kalimantan, Laos, Myanmar, Pakistan, Philippines, Ryukyu Is, Sri Lanka, Thailand, Vietnam, Andaman Is, Mauritius
B. acuruana Moric.	Brazil (Bahia, Serra Açuruá, Minas Gerais, S. Romão)
B. affinis Vogel	Brazil (Santa Catarina)
B. albicans Vogel	Brazil (Rio de Janeiro)
B. angulicaulis Harms	Brazil (Goiás)
B. andrieuxii Hemsl.	Mexico
B. ankarafantsikae Du Puy et R. Rabev.	Madagascar
B. argentinensis Burkart<br>(= *B. megasiphon* Burkart)	Argentina, Paraguay
B. augustii Harms	Ecuador, Peru
B. aurantiaca Bojer	Comoro Is, Madagascar, Mauritius
B. aureopunctata Ducke	Brazil (Pará, Vila Braga, lugar Francez, Bella Vista, Tapajós River)
B. baina J.F. Macbr.	Peru (Río Mazán)
B. bauhinioides (Mart.) J.F. Macbr.<br>(= *B. microphylla* Vogel)	Caribbean-TRP, Argentina, Brazil (Minas Gerais, Mato Grosso, Bahia, Ceará, Piauí, Maranhão), Paraguay, Venezuela
B. beguinotii Cufod.	Costa Rica, Panama, Colombia
B. bicolor (Bong.) Steud.	Brazil
B. blakeana Dunn	China (Guangdong), India, Indonesia-ISO, Malaysia-ISO, Papua New Guinea, Mauritius, Reunion, Rodrigues, Northern Marianas
B. bohniana L. Chen	China (Yunnan)
B. bombaciflora Ducke	Brazil (Pará, Itaboca, Tocantins River)
B. bowkeri Harv.	South Africa, Zimbabwe, Australia
B. brachycalyx Ducke	Brazil (Amazonas), Ecuador

B. brachycarpa Benth.	China (Gansu, Guangxi, Guizhou, Hubei, Sichuan, Xizang, Yunnan), Laos, Myanmar, Thailand
B. brevicalyx Du Puy et R. Rabev.	Madagascar
B. brevipes Vogel<br>(= *B. bongardii* Steud.)	Bolivia, Brazil (Minas Gerais, Pitangui)
B. burchellii Benth.	Brazil (Goiás)
B. burrowsii E.J.D. Schmidt [15]	Mozambique
B. buscalionii Mattei	Somalia
B. calliandroides Rusby	Bolivia
B. caloneura Malme	Brazil (Mato Grosso, Cuiabá)
B. campestris Malme	Brazil (Mato Grosso, Chapada dos Guimarães)
B. candelabriformis R.S. Cowan	Brazil (Goiás)
B. capuronii Du Puy et R. Rabev.	Madagascar
B. catingae Harms	Brazil (Bahia, Tamburi)
B. chalkos R.S. Cowan	Venezuela
B. chapulhuacania Wunderlin	Mexico
B. cheilantha (Bong.) Steud.	Brazil (Mato Grosso, Ceará, Serra de Maranguape, Riacho Pirapora)
B. cinnamomea DC.	Brazil (Guayana Francesa, Cayenna), French Guiana, Guyana, Peru, Surinam
B. conwayi Rusby<br>(= *B. straussiana* Harms, *B. tumupasensis* Rusby)	Bolivia (Tumupasa, Gebiet des Alto Acre, Cobija), Brazil, Peru
B. cookii Britton et Rose	Costa Rica, Guatemala, Honduras, Mexico
B. corniculata Benth.	Bolivia, Brazil (Pará, Óbidos), Colombia, Peru
B. coulteri J.F. Macbr.	Mexico
B. cupulata Benth.	Brazil (Piauí, Goiás), Venezuela
B. curvula Benth.	Brazil (Goiás, Goiás Velho)
B. darainensis Thulin, L. Nusbaumer et L. Gautier [50]	Madagascar (Loky-Manambato)
B. decandra Du Puy et R. Rabev.	Madagascar
B. dipetala Hemsl.	Caribbean-TRP, Belize, Guatemala, Mexico
B. divaricata L.	Cayman Is, Cuba, Dominican Republic, Haiti, Jamaica, St Kitts-Nevis, Virgin Is, Belize, Costa Rica, El Salvador, Guatemala, Honduras, Mexico, Nicaragua, United States (Texas), Mauritius
B. dubia G. Don<br>(= *B. viridiflora* Ducke, *B. nitida* Benth.,<br>non *Bauhinia dubia* Vogel)	Brazil (Pará, Tocantins, Pará, Alcobaça Railway, Breu Branco Station, Tocantins river)
B. dumosa Benth.	Brazil (Goiás, Bahia, Juazeiro, São Francisco River, Minas Gerais)
B. eilertsii Pulle	Surinam
B. ellenbeckii Harms	Ethiopia, Somalia, Yemen
B. elongipes R.S. Cowan	Brazil (Goiás, Formosa)

B. erythrocalyx Wunderlin	Guatemala, Mexico
B. estrellensis Hassl.	Paraguay
B. exellii Torre et Hillc.	Angola-ISO
B. farek Desv.	Ethiopia
B. flagelliflora Wunderlin	Ecuador
B. forficata Link<br>(= *B. armata* Thunb., *B. aculeata* Vell., non *B. aculeata* L.)	Tonga, Argentina, Bolivia, Brazil (São Paulo, Amparo), Paraguay, Peru, Uruguay
B. foveolata Dalzell	India (Gujarat, Karnataka, Maharashtra)
B. fryxellii Wunderlin	Mexico
B. fusconervis (Bong.) Steud.<br>(= *B. selloviana* Vogel)	Brazil (Minas Gerais)
B. galpinii N.E. Br.	Ghana, Kenya, Malawi, Mozambique, South Africa, Swaziland, Zambia, Zimbabwe, India (Karnataka, Maharashtra, Punjab, Tamil Nadu, West Bengal), Indonesia-ISO, Malaysia-ISO, Philippines, Sri Lanka, Norfolk Is, Caribbean-TRP, Mauritius, Reunion, Rodrigues, Seychelles, Fiji
B. gardneri Benth.	Brazil (Goiás)
B. gilesii F. Muell. et Bailey	Australia (Western Australia)
B. glaziovii Taub.	Brazil
B. goyazensis Harms	Brazil (Goiás, Fazenda do Paranana)
B. grandidieri Baill.	Madagascar
B. grandifolia (Bong.) Steud.	Brazil (Amazonas)
B. grevei Drake	Madagascar
B. gypsicola McVaugh	Mexico
B. hagenbeckii Harms<br>(= *B. hassleriana* Chodat)	Brazil, Paraguay
B. haughtii Wunderlin	Ecuador
B. hildebrandtii Vatke	Madagascar
B. hirsuta Weinm.<br>(= *B. parvula* Gagnep.)	Cambodia, China (Yunnan), Indonesia-ISO, Java, Laos, Malaysia-ISO, Peninsular Malaysia, Thailand, Vietnam
B. holophylla (Bong.) Steud.<br>(= *B. cordata* Vogel, *B. dodecandra* (Bong.) Steud.)	Brazil (Mato Grosso, Minas Gerais)
B. humilis Rusby	Bolivia
B. integerrima Benth.<br>(= *B. odoratissima* Moric.)	Brazil (Bahia)
B. jenningsii P. Wilson	Caribbean-TRP, Belize, Guatemala, Mexico
B. jucunda Brandegee	Mexico
B. kalantha Harms	Tanzania
B. lamprophylla Harms	Brazil (Goiás)
B. leptantha Malme	Brazil (Mato Grosso, Corumbá)

B. leucantha Thulin	Somalia
B. longicuspis Benth. [25]<br>(= *B. stenocardia* Standl.)	Bolivia, Brazil (Amazonas, Pará, Aramanahy, Lower Tapajóz), Peru, Venezuela
B. longifolia (Bong.) Steud.<br>(= *B. geminata* Vogel, *B. obtusata* Vogel, *B. recurva* R.S. Cowan)	Bolivia, Brazil (Mato Grosso, Goiás, São João da Aliança), Paraguay, Peru
B. longipedicellata Ducke	Brazil (Pará)
B. macrantha Oliv.	Angola (Ninda)
B. macranthera Hemsl.	Mexico, United States (Florida, Texas)
B. madagascariensis Desv.<br>(= *B. commersonii* Decne.)	Madagascar, Mauritius
B. malacotricha Harms	Brazil (Goiás, Chapadão dos Veadeiros)
B. malacotrichoides R.S. Cowan	Brazil (Goiás)
B. marginata (Bong.) Steud.	Brazil (São Paulo)
B. martinensis J.F. Macbr.	Peru (San Martín)
B. membranacea Benth.	Brazil (Goiás)
B. mendoncae Torre et Hillc.	Angola-ISO, Zambia
B. mollis (Bong.) D. Dietr.<br>(= *B. corumbensis* S. Moore)	Argentina, Bolivia, Brazil (Mato Grosso, Cuiabá, Camapuã), Paraguay
B. mombassae Vatke<br>(= *B. loesneriana* Harms)	Kenya
B. monandra Kurz<br>(= *B. porosa* Boivin ex Baill., *B. punctiflora* Baker)	Angola-ISO, Burundi, Ghana, Ivory Coast, Liberia, Mali, Nigeria, Sierra Leone, Somalia, Tanzania, Zaire, Zambia, Bangladesh, Myanmar, India (Karnataka, Maharashtra, Tamil Nadu, West Bengal), Indonesia-ISO, Malaysia-ISO, Sri Lanka, Thailand, Vietnam, Papua New Guinea, Caribbean-TRP, Mexico, Panama, Madagascar, Rodrigues, United States, Cook Is, Australia (naturalized), Fiji, Niue, Northern Marianas, Society Is, Tonga, Brazil, Colombia, French Guiana
B. morondavensis Du Puy et R. Rabev.	Madagascar
B. multinervia (Kunth) DC.	Caribbean-TRP, Brazil, Surinam, Venezuela
B. natalensis Hook.	South Africa
B. ombrophila Du Puy et R. Rabev.	Madagascar
B. ovata (Bong.) Vogel	Brazil
B. pansamalana Donn. Sm.	Guatemala, Mexico
B. pauletia Pers.	Caribbean-TRP, Costa Rica, El Salvador, Guatemala, Honduras, Mexico, Nicaragua, Panama, Colombia, Venezuela
B. pentandra (Bong.) D. Dietr.	Brazil (Mato Grosso, Goiás, Goiás Velho, Piauí, Boa Esperança, Bahia, Joazeiro, Ceará, near Icó)
B. pervilleana Baill.	Madagascar
B. pes-caprae Cav.	Mexico

B. petersiana Bolle	Angola-ISO, Botswana, Malawi, Mozambique, Namibia-ISO, South Africa, Tanzania, Zaire, Zambia, Zimbabwe, India (West Bengal)
B. petiolata (DC.) Hook.	Panama, Colombia, Venezuela
B. pichinchensis Wunderlin	Ecuador
B. picta (Kunth) DC.	Panama, Colombia, Venezuela
B. pinheroi Wunderlin	Brazil (Bahia, Fazenda Pau-Brazil)
B. platypetala Benth.	Bolivia, Brazil [Tocantins, Porto Real (currently Porto Nacional), Goiás]
B. platyphylla Benth.	Brazil (Goiás)
B. podopetala Baker	Madagascar
B. pottsii G. Don	Myanmar, Cambodia, Indonesia-ISO, Java, Kalimantan, Malaysia-ISO, Peninsular Malaysia, Sumatra, Thailand
B. prainiana Craib	Myanmar, Thailand
B. pringlei S. Watson	Mexico
B. pulchella Benth.	Brazil (Piaui, Oeiras, Minas Gerais, Minas Novas, Bahia, Serra Açurua)
B. purpurea L.	Ethiopia, Malawi, Mozambique, Nigeria, Sierra Leone, South Africa, Uganda, Zaire, Zambia, Bangladesh, Bhutan, Brunei, China (Hainan, Hong Kong, Taiwan), India (Andhra Pradesh, Arunachal Pradesh, Assam, Bihar, Delhi, Goa, Gujarat, Haryana, Himachal Pradesh, Jammu-Kashmir, Karnataka, Kerala, Madhaya Pradesh, Maharashtra, Manipur, Meghalaya, Mizoram, Nagaland, Orissa, Pondicherry, Punjab, Rajasthan, Sikkim, Tamil Nadu, Tripura, Uttar Pradesh, West Bengal), Indonesia-ISO, Iraq, Laos, Malaysia-ISO, Myanmar (Yanan), Nepal, Pakistan, Philippines, Ryukyu Is, Sri Lanka, Thailand, Vietnam, Papua New Guinea, Barbados, Martinique, Puerto Rico, St Lucia, Costa Rica, El Salvador, Guatemala, Mexico, Panama, Andaman Is, Madagascar, Mauritius, Nicobar Is, United States, Fiji, Northern Marianas, Pitcairn Is
B. racemosa Lam.<br>(= *B. parviflora* Vahl)	Mauritania, Sierra Leone, Tanzania, Uganda, Bangladesh, Cambodia, China (Yunnan), India (Andhra Pradesh, Arunachal Pradesh, Assam, Bihar, Dadra-Nagar-Haveli, Goa, Gujarat, Haryana, Himachal Pradesh, Karnataka, Kerala, Madhaya Pradesh, Maharashtra, Manipur, Meghalaya, Orissa, Pondicherry, Punjab, Rajasthan, Tamil Nadu, Tripura, Uttar Pradesh, West Bengal), India-ISO, Myanmar,

	Pakistan, Sri Lanka, Thailand, Vietnam, Dominica, Mauritius
B. ramosissima Hemsl.	Mexico
B. richardiana DC.	Zaire, French Guiana
B. roxburghiana Voigt<br>(= *B. emarginata* G. Don)	India (Sikkim, Uttar Pradesh, West Bengal), Nepal
B. rubeleruziana Donn. Sm.	Belize, Guatemala, Mexico
B. rufa (Bong.) Steud.<br>(= *B. intermedia* Vogel, *B. choriophylla* Vogel)	Brazil (Minas Gerais, Rio de Janeiro)
B. rufescens Lam.	Benin, Cameroon, Chad, Ghana, Guinea, Guinea Bissau, Ivory Coast, Mali, Mauritania, Niger, Nigeria, Senegal, Sierra Leone, Sudan, Togo, India (Pondicherry, Tamil Nadu, West Bengal), India-ISO
B. rusbyi Britton	Bolivia
B. saccocalyx Pierre	Laos, Thailand
B. seleriana Harms	Guatemala, Honduras, Mexico
B. seminarioi Eggers	Ecuador
B. sessilifolia (DC.) Quinones	Columbia
B. smilacifolia Burch. ex Benth.	Brazil (Goiás, Tocantins)
B. somalensis Pic. Serm. et Roti Mich.	Somalia
B. stenantha Diels	Ecuador
B. strychnifolia Craib	Thailand
B. subclavata Benth.	Brazil (Piauí)
B. subrotundifolia Cav.	Mexico
B. taitensis Taub.	Kenya
B. tarapotensis Benth.<br>(= *B. amplifolia* Ducke)	Brazil (Amazonas, Tabatinga, Solimões River), Colombia, Ecuador, Peru (San Martin, Tarapoto)
B. tenella Benth.	Brazil (Tocantins, Arraias, Goiás)
B. tomentosa L.	Angola-ISO, Cameroon, Ethiopia, Ghana, Kenya, Nigeria, Sierra Leone, Somalia, South Africa, Tanzania, Zaire, Zambia, Zimbabwe, Bangladesh, China (Guangdong, Hong Kong), India (Andhra Pradesh, Bihar, Delhi, Goa, Gujarat, Haryana, Himachal Pradesh, Jammu-Kashmir, Karnataka, Kerala, Madhaya Pradesh, Maharashtra, Orissa, Pondicherry, Punjab, Rajasthan, Tamil Nadu, Uttar Pradesh, West Bengal), Myanmar (Yanan), Indonesia-ISO, Malaysia-ISO, Nepal, Pakistan, Sri Lanka, Thailand, Vietnam, Papua New Guinea, Antigua-Barbuda, Barbados, Cuba, Dominica, Dominican Republic, Guadeloupe, Martinique, Montserrat, Puerto Rico, St Kitts-Nevis, St Lucia, Mauritius, Fiji, Northern Marianas, Tonga, Brazil

B. tortuosa Collett et Hemsl.	Myanmar
B. tuichiensis Cayola et A. Fuentes [14]	Bolivia
B. ungulata L.<br>(= *B. macrostachya* Benth.)	Belize, Costa Rica, El Salvador, Guatemala, Mexico, Nicaragua, Panama, Seychelles, Bolivia, Brazil, Colombia, Paraguay, Venezuela
B. urbaniana Schinz	Angola-ISO, Botswana, Namibia-ISO, Zambia
B. urocalyx Harms	Bolivia, Brazil (Acre, Juruá-Mirim river, Porto Walter), Peru
B. uruguayensis Benth.	Argentina (Corrientes, Misiones), Brazil (Parana, Rio Grande do Sul, Santa Catarina, Sao Paulo), Paraguay
B. variegata L.	Ethiopia, Ghana, Kenya, Malawi, Mozambique, Nigeria, Sierra Leone, South Africa, Tanzania, Uganda, Zaire, Zambia, Zimbabwe, Bangladesh, Bhutan, China (Hainan, Hong Kong), India (Andhra Pradesh, Arunachal Pradesh, Assam, Delhi, Goa, Gujarat, Haryana, Himachal Pradesh, Jammu-Kashmir, Karnataka, Kerala, Madhaya Pradesh, Maharashtra, Manipur, Meghalaya, Mizoram, Nagaland, Orissa, Pondicherry, Punjab, Rajasthan, Sikkim, Tamil Nadu, Tripura, Uttar Pradesh, West Bengal), India-ISO, Indonesia-ISO, Iraq, Laos, Malaysia-ISO, Myanmar (Yanan), Nepal, Pakistan, Sri Lanka, Thailand, Vietnam, Norfolk Is, Papua New Guinea, Bahamas, Dominican Republic, Grenada, Haiti, Puerto Rico, St Lucia, El Salvador, Mexico, Panama, Mauritius, Seychelles, United States (California, Florida, Texas), Fiji, New Zealand, Society Is, Tonga, Brazil, Colombia
B. vespertilio S. Moore	Brazil (Mato Grosso, Santa Cruz)
B. weberbaueri Harms<br>(= *B. ruiziana* Harms)	Ecuador, Peru
B. xerophyta Du Puy et R. Rabev.	Madagascar

Table 2 The species and distribution of *Phanera* Lour.

**Accepted names (92 in number)**	**Distribution**
P. aherniana (Perkins) de Wit	The Philippines, Sabah
P. andersonii (K. Larsen et S.S. Larsen) Bandyop. et al. [9]	Peninsular Malaysia
P. argentea de Wit<br>(= *B. argentea* (de Wit) Cusset, *B. endertii* K. Larsen et S.S. Larsen)	Sarawak, Sabah, Kalimantan, Indonesia-ISO, Malaysia-ISO
P. audax de Wit<br>(= *Bauhinia calycina* Gagnep.)	Cambodia, Malaysia-ISO, Peninsular Malaysia
P. aurea (H. Lév.) Mackinder et R. Clark	China
P. aureifolia (K. Larsen et S.S. Larsen) Bandyop. et al. [9]	Thailand

P. bassacensis (Gagnep.) de Wit	Peninsular Malaysia, Thailand, Cambodia, Laos, Vietnam, Sabah, Java
P. bidentata (Jack) Benth.	Peninsular Malaysia, Sumatra
P. bracteata Benth.	Myanmar, Thailand, Cambodia, Laos, Vietnam
P. brevipedicellata (Jarvie) Mackinder et R. Clark [16]	Kalimantan (= Borneo)
P. burbidgei (Stapf) Bandyop.et al.	Sarawak, Brunei, Sabah
P. calciphylla (D.X. Zhang et T.C. Chen) Mackinder et R. Clark	China
P. campanulata (S.S. Larsen) Bandyop. et al.	Brunei
P. carcinophylla (Merr.) Mackinder et R. Clark	China, Vietnam
P. chalcophylla (L. Chen) Mackinder et R. Clark	China
P. clemensiorum (Merr.) Bandyop. et al.<br>(= *Bauhinia clemensorum* Gagnep.)	Vietnam
P. coccinea Lour.	China, Laos, Vietnam
P. crudiantha de Wit	Sabah
P. cuprea (Ridl.) de Wit	Peninsular Malaysia
P. damiaoshanensis (T.C. Chen) comb. nov. [this paper]<br>Basionym: *Bauhinia damiaoshanensis* T.C. Chen in *Guihaia* 8: 49. 1988 [37]	China (Guangxi)
P. decumbens (M.R. Hends) de Wit	Peninsular Malaysia
P. didyma (L. Chen) comb. nov. [this paper]<br>Basionym: *Bauhinia didyma* L. Chen in *J Arnold Arbor* 19: 131. 1938 [38]	China (Guangdong, Guangxi)
P. diphylla (Buch. - Ham.) Benth.	India, Sri Lanka, Myanmar
P. divergens (Baker) Thoth.	India, Myanmar
P. elmeri (Merr.) de Wit	Sarawak, Sabah, Kalimantan, Indonesia-ISO, Malaysia-ISO
P. erythropoda (Hayata) Mackinder et R. Clark	China
P. excelsa Miq.	Sarawak, Brunei, Sabah, Kalimantan
P. excurrens (Stapf) Bandyop. et al.	Sabah
P. fabrilis (de Wit) Bandyop. et al.	Sabah
P. ferruginea (Roxb.) Benth.	Peninsular Malaysia, Thailand, Sumatra, Indonesia-ISO, Malaysia-ISO, Myanmar, Nicobar Is
P. finlaysoniana Benth.	Sumatra, Sarawak, Sabah, Kalimantan, Sulawesi, Philippines, Molucca Is
P. foraminifera (Gagnep.) de Wit	Brunei, Malaysia-ISO, Sarawak
P. franckii (K. Larsen et S.S. Larsen) Bandyop. et al.	Peninsular Malaysia
P. fulva (Korth.) Benth.	Sumatra, Java
P. glabrifolia Benth.	Bhutan, India, Myanmar, Peninsular Malaysia, Thailand, Laos
P. glabristipes de Wit	Malaysia-ISO, Sarawak
P. gracillima de Wit	Sabah

P. hainanensis (Merr. et Chun ex L. Chen) comb. nov. [this paper]<br>Basionym: *Bauhinia hainanensis* Merr. et Chun ex L. Chen in *J Arnold Arbor* 19: 132. 1938 [38]	China (Hainan)
P. havilandii (Merr.) Bandyop. et al.	Sarawak, Brunei, Sabah, Kalimantan
P. hekouensis (T.Y. Tu et D.X. Zhang) comb. nov. [this paper]<br>Basionym: *Bauhinia hekouensis* T.Y. Tu et D.X. Zhang in *Novon* 22: 332. 2013 [2]	China (Yunnan)
P. hendersonii de Wit	The Anambas Is, Indonesia
P. hypochrysa (T.C. Chen) Mackinder et R. Clark	China
P. integrifolia (Roxb.) Benth.	Peninsular Malaysia, Thailand, Sabah, Sumatra, the Philippines
P. involucellata (Kurz) de Wit<br>(= *Bauhinia involucellata* Kurz)	Myanmar, Thailand
P. involucrans (Gagnep.) comb. nov. [this paper]<br>Basionym: *Bauhinia involucrans* Gagnep.in *Bull Mus Natl Hist Nat Sér* 2, 24: 313. 1952 [40]	Vietnam
P. jampuiensis Darlong et D. Bhattach. [18]	India
P. khasiana (Baker) Thoth.	China (Hainan), India, Thailand, Laos, Vietnam
P. kingii (Prain) Bandyop. et al.	Peninsular Malaysia
P. kockiana (Korth.) Benth.	Peninsular Malaysia, Sumatra, Sarawak, Brunei, Sabah, Kalimantan, the Lesser Sunda Is
P. kurzii (Prain) Thoth.	Myanmar
P. kostermansii (K. Larsen et S.S. Larsen) Bandyop. et al.	Sabah, Kalimantan
P. lambiana (Baker f.) de Wit	Sarawak, Brunei
P. lingua (DC.) Miq.	Sulawesi, Lesser Sunda Is, Philippines, Papua New Guinea
P. lorantha (Pierre ex Gagnep.) comb. nov. [this paper]<br>Basionym: *Bauhinia lorantha* Pierre ex Gagnep. in *Notul Syst (Paris)* 2: 175. 1912 [41]	Laos
P. lucida Benth.	Peninsular Malaysia, Sumatra
P. lyrata (Raizada) Thoth.	Myanmar
P. meeboldii (Craib) Thoth.	Myanmar
P. menispermacea (Gagnep.) de Wit	Sarawak, Kalimantan
P. merrilliana (Perkins) de Wit	Sarawak, Sabah, the Philippines
P. nakhonphanomensis (Chatan) Mackinder et R. Clark<br>(= *Bauhinia nakhonphanomensis* Chatan) [3]	Thailand
P. nervosa Benth.	China (Yunnan), India, Bangladesh, Myanmar, Thailand
P. ornata (Kurz) Thoth.	China, India, Myanmar, Thailand, Laos, Vietnam
P. ovatifolia (T.C. Chen) comb. nov. [this paper]	China (Guangxi), India (Arunachal Pradesh)

Basionym: *Bauhinia ovatifolia* T.C. Chen in *Guihaia* 8: 50. 1988 [37]	
P. pachyphylla (Merr.) de Wit	The Philippines
P. pauciflora (Merr.) de Wit	The Philippines
P. paucinervata (T.C. Chen) X.Y. Zhu [46]<br>(*P. paucinervata* (T.C. Chen) Mackinder et R. Clark, *nom. inval.*)	China (Guangxi)
P. phoenicea (Wight et Arn.) Benth.	India
P. posthumi de Wit	Sumatra
P. pottingeri (Prain) Thoth.	Myanmar
P. praesignis (Ridl.) de Wit	Peninsular Malaysia
P. pyrrhoclada (Drake) de Wit	China, Vietnam
P. pyrrhoneura (Korth.) Benth.	Sumatra
P. quinanensis (T.C. Chen) comb. nov. [this paper]<br>Basionym: *Bauhinia quinanensis* T.C. Chen in *Guihaia* 8: 48. 1988 [37]	China (Guizhou)
P. rubro-villosa (K. Larsen et S.S. Larsen) Mackinder et R. Clark	China, Laos, Vietnam
P. rahmatii (Merr.) Bandyop. et al.	Sumatra
P. ridleyi (Prain) A. Schmitz.	Peninsular Malaysia, Thailand
P. saigonensis (Pierre ex Gagnep.) Mackinder et R. Clark	Thailand, Cambodia, Laos, Vietnam
P. semibifida (Roxb.) Benth.	Peninsular Malaysia, Sumatra, Sarawak, Sabah, Kalimantan, Sulawesi, the Philippines
P. siamensis (K. Larsen et S.S. Larsen) Mackinder et R. Clark<br>(= *Bauhinia siamensis* K. Larsen et S.S. Larsen) [4]	Thailand
P. similis (Craib) de Wit	Myanmar, Thailand, Laos
P. sirindhorniae (K. Larsen et S.S. Larsen) Mackinder et R. Clark	Thailand
P. stipularis (Korth.) Benth.	India, the Nicobar Islands, Sumatra
P. steenisii (K. Larsen et S.S. Larsen) Bandyop. et al.	Sabah
P. sulphurea (C.E.C. Fischer) Thoth.	Myanmar
P. sylvani de Wit	Sarawak, Sabah
P. tianlinensis (T.C. Chen et D.X. Zhang) Mackinder et R. Clark	India, Bhutan
P. vahlii (Wight et Arn.) Benth.	India, Bhutan
P. wallichii (J.F. Macbr.) Thoth.<br>(= *Bauhinia wallichii* J.F. Macbr., *B. melanophylla* Merr., *Phanera macrostachya* Benth.)	India, Myanmar, Thailand, Vietnam, China (Yunnan)
P. williamsii (F. Muell.) de Wit	Papua New Guinea, Australia
P. wrayi (Prain) de Wit	Peninsular Malaysia, Sumatra, Sarawak, Brunei, Sabah, Kalimantan
P. wuzhengyii (S.S. Larsen) Bandyop. et al.<br>(= *Bauhinia wuzhengyii* S.S. Larsen) [5]	China
P. yunnanensis (Franch.) Wunderlin<br>(= *Bauhinia yunnanensis* Franch., *Phanera collettii* Thoth.)	China, Myanmar, Thailand

Table 3 The species and distribution of *Schnella* Raddi

**Accepted names (49 in number)**	**Distribution**
S. accrescens (Killip et J.F. Macbr.) Trethowan et R. Clark [47]	Peru (Mishuyacu)

(= *Bauhinia accrescens* Killip et J.F. Macbr.) [35]	
S. alata (Ducke) Wunderlin	Brazil
S. altiscandens (Ducke) Wunderlin<br>(= *Bauhinia stenopetala* Ducke, *S. stenopetala* (Ducke) Wunderlin)	Brazil
S. anamesa (J.F. Macbr.) Wunderlin	Brazil (Goias and Mato Grosso), Caribbean-TRP, Northern Marianas
S. angulosa (Vogel) Wunderlin	Brazil
S. bahiachalensis Zamora [48]	Costa Rica
S. carvalhoi (Vaz) Wunderlin	Brazil
S. confertiflora (Benth.) Wunderlin	Brazil
S. coronata (Benth.) Pittier<br>(= *Bauhinia coronata* Benth., *B. dubia* Vogel)	Bolivia, Brazil, French Guiana
S. cupreonitens (Ducke) Wunderlin	Brazil, Peru
S. erythrantha (Ducke) Wunderlin	Brazil
S. excisa Grisebach [47]	Panama, Trinidad & Tobago
S. flexuosa (Moric.) Walp.	Brazil
S. glabra (Jacq.) Dugand.	Caribbean-TRP, Belize, Costa Rica, Mexico, Panama, Brazil, Colombia, Ecuador, French Guiana, Peru, Venezuela
S. grazielae (Vaz) Wunderlin	Brazil
S. guentheri (Harms) Trethowan et R. Clark [47]<br>(= *Bauhinia guentheri* Harms) [44]	Peru (Mouth of Río Santiago)
S. guianensis (Aubl.) Wunderlin [10]<br>(= *Bauhinia guianensis* Aubl.)	Dominica, Guadeloupe, Belize, Costa Rica, Mexico, Panama, Bolivia, Brazil, Colombia, Ecuador, French Guiana, Guyana, Peru, Surinam, Venezuela
S. herrerae Britton et Rose	Belize, Guatemala, Mexico
S. hirsutissima (Wunderlin) Trethowan et R. Clark [47]<br>(= *Bauhinia hirsutissima* Wunderlin) [31]	Peru
S. hymenaeifolia (Hemsl.) Britton et Rose	Panama
S. kleiniana (Burkart) comb. nov. [this paper]<br>Basionym: *Bauhinia kleiniana* Burkart in *Darwiniana* 12: 251. 1961 [34]	Brazil (Santa Catarina, Blumenau, Morro Spitzkopf)
S. klugii (Standl.) Wunderlin<br>(= *Bauhinia klugii* Standl., *Phanera klugii* (Standl.) Vaz)	Peru
S. kunthiana (Vogel) Wunderlin<br>(= *Bauhinia kunthiana* Vogel, *Phanera kunthiana* (Vogel) Vaz)	Brazil, French Guiana, Guyana, Surinam, Venezuela
S. lilacina (Wunderlin et Eilers) Wunderlin<br>(= *Bauhinia lilacina* Wunderlin et Eilers)	Brazil
S. longiseta (Ducke) Wunderlin	Brazil (Amazonas)
S. macrostachya Raddi<br>(= *Bauhinia radiata* Vell., *Phanera radiata* (Vell.) Vaz [24], *S. radiata* (Vell.) Trethowan et R. Clark [47])	Brazil (Minas Gerais, Rio de Janeiro)
S. maximilianii (Benth.) Wunderlin	Brazil

S. microstachya Raddi<br>(= *Phanera microstachya* (Raddi) L.P. Queiroz) [6]	Belize, Guatemala, Mexico, Panama, Argentina, Bolivia, Brazil, Colombia, Ecuador, Paraguay, Peru (San Martín), Venezuela
S. obovata (S.F. Blake) Britton et Rose [47]<br>(= *Bauhinia obovata* S.F. Blake)	Panama
S. outimouta (Aubl.) Wunderlin	Brazil
S. platycalyx (Benth.) Wunderlin	Brazil
S. poiteauana (Vogel) Wunderlin	Brazil, French Guiana, Surinam
S. porphyrotricha (Harms) Wunderlin	Brazil, Peru
S. pterocalyx (Ducke) Wunderlin	Brazil
S. reflexa (Schery) Wunderlin	Panama, Columbia
S. riedeliana (Bong.) Wunderlin	Brazil (Mato Grosso do Sul, Sao Paulo)
S. rutilans (Spruce ex Benth.) Pittier	Brazil, Ecuador, Peru, Venezuela
S. scala-simiae (Sandwith) Trethowan et R. Clark [47]<br>(= *Bauhinia scala-simiae* Sandwith)	Guyana, Venezuela
S. siqueirae (Ducke) Wunderlin	Brazil, Guyana
S. smilacina G. Don	Brazil (Espírito Santo, Rio de Janeiro)
S. splendens Benth.	Venezuela
S. sprucei (Benth.) Wunderlin	Brazil
S. stenoloba Britton et Killip [47]	Colombia
S. surinamensis (Amshoff) Wunderlin	Brazil, Guyana, Surinam, Venezuela
S. tessmannii (Harms) comb. nov. [this paper]<br>Basionym: *Bauhinia tessmannii* Harms in Mildbraed, *Notizbl Bot Gart Berlin-Dahlem* 9: 261. 1925 [43]	Peru (Cachibo Playa)
S. trichosepala (L.P. Quieroz) Wunderlin	Brazil (Bahia, Minas Gerais)
S. uleana (Harms) Wunderlin	Brazil, Peru
S. vestita Benth. [47]	Colombia
S. vulpina (Rusby) Trethowan et R. Clark [47]<br>(= *Bauhinia vulpina* Rusby)	Bolivia

Table 4 The species and distribution of *Lasiobema* (Korth.) Miq.

**Accepted names (22 in number)**	**Distribution**
L. apertilobata (Merr. et F.P. Metcalf) com. nov. [this paper]<br>Basionym: *Bauhinia apertilobata* Merr. et F.P. Metcalf in *Lingnan Sci J* 16: 83. 1937 [45]	China (Fujian, Guangdong, Guangxi, Jiangxi)
L. cardinale (Pierre ex Gagnep.) de Wit [12]<br>(= *Bauhinia cardinalis* Pierre ex Gagnep., *B. dolichobotrys* Merr.)	Cambodia, Laos, Vietnam
L. championii (Benth.) de Wit [12]<br>(= *Phanera championii* Benth., *Bauhinia championii* (Benth.) Benth., *B. esquirolii* Gagnep.)	China (Hainan, Fujian, Guangdong, Guangxi, Guizhou, Hubei, Hunan, Jiangxi, Zhejiang, Hong Kong, Taiwan), India (Assam, Sikkim, West Bengal), Vietnam
L. comosa (Craib) A. Schmitz [36]	China (Sichuan, Yunnan)

(= *Bauhinia comosa* Craib, *B. henryi* Harms)	
L. concreta (Craib) A. Schmitz<br>[lapsus calami: *L. concreta* (Prain) A. Schmitz] [36]<br>(= *Bauhinia concreta* Craib)	Thailand
L. curtisii (Prain) de Wit [12]	Cambodia, Laos, Malaysia-ISO, Peninsular Malaysia, Thailand, Vietnam
L. delavayi (Franch.) A. Schmitz [36]<br>(= *Bauhinia delavayi* Franch.)	China (Yunnan)
L. flavum de Wit<br>(= *Bauhinia flava* (de Wit) Cusset)	Malaysia-ISO, Peninsular Malaysia
L. godefroyi (Gagnep.) com. nov. [this paper]<br>Basionym: *Bauhinia godefroyi* Gagnep. in *Notul Syst (Paris)* 2: 278. 1912 [42]	Cambodia
L. harmsianum (Hosseus) de Wit<br>(= *Bauhinia harmsiana* Hosseus)	Cambodia, Thailand
L. hypoglauca (T.C. Chen) com. nov. [this paper]<br>Basionym: *Bauhinia hypoglauca* T.C. Chen in *Guihaia* 8: 44. 1988 [37]	China (Yunnan)
L. japonicum (Maxim.) de Wit	China (Guangdong), Japan (Ryukyu Is)
L. lingyuenensis (T.C. Chen) comb. nov. [this paper]<br>Basionym: *Bauhinia lingyuenensis* T.C. Chen in *Guihaia* 8: 45. 1988 [37]	China (Guangxi)
L. longistipes (T.C. Chen) comb. nov. [this paper]<br>Basionym: *Bauhinia longistipes* T.C. Chen in *Guihaia* 8: 43. 1988 [37]	China (Yunnan)
L. oxysepala (Gagnep.) com. nov. [this paper]<br>Basionym: *Bauhinia oxysepala* Gagnep. in *Notul Syst (Paris)* 2: 176. 1912 [41]	Vietnam
L. penicilliloba (Pierre ex Gagnep.) de Wit [12]<br>(= *Bauhinia penicilliloba* Pierre ex Gagnep.)	Cambodia, Laos, Thailand, Vietnam
L. pulla (Craib) A. Schmitz [36]<br>(= *Bauhinia pulla* Craib)	Cambodia, Thailand
L. retusa (Roxb.) de Wit [12]	Bangladesh, Nepal, Pakistan, India (Andhra Pradesh, Bihar, Haryana, Himachal Pradesh, Karnataka, Madhaya Pradesh, Maharashtra, Orissa, Punjab, Rajasthan, Tamil Nadu, Uttar Pradesh, West Bengal)
L. scandens (L.) de Wit [12]	Bangladesh, Bhutan, Cambodia, China (Hainan), India (Andhra Pradesh, Arunachal Pradesh, Assam, Bihar, Goa, Gujarat, Karnataka, Kerala, Madhaya Pradesh, Maharashtra, Meghalaya, Mizoram, Orissa, Pondicherry, Sikkim, Tamil Nadu, Tripura, West Bengal), Indonesia-ISO, Java, Laos, Lesser Sunda Is, Myanmar, Nepal, Sri Lanka, Sumatra, Thailand, Vietnam
L. strychnoidea (Prain) de Wit [12]	Malaysia-ISO, Peninsular Malaysia

L. tubicalyx (Craib) de Wit [12]	Thailand
L. venustula (T.C. Chen) comb. nov. [this paper]<br>Basionym: *Bauhinia venustula* T.C. Chen in *Guihaia* 8: 45. 1988 [37]	China (Guangxi)

Table 5 The species and distribution of *Gigasiphon* Drake

**Accepted names (6 in number)**	**Distribution**
G. amplum (Span.) de Wit	East Timor, Indonesia-ISO, Irian Jaya, Papua New Guinea
G. dolichocalyx (Merr.) de Wit	The Philippines
G. gossweileri (Baker f.) Torre et Hillc.	Angola-ISO, Gabon, Zaire
G. humblotianum (Baill.) Drake<br>(= *Bauhinia humblotiana* Baill.)	Madagascar
G. macrosiphon (Harms) Brenan	Kenya, Tanzania
G. schlechteri (Harms) de Wit	Indonesia-ISO, Irian Jaya, Papua New Guinea

Table 6 The species and distribution of *Piliostigma* Hochst.

**Accepted names (3 in number)**	**Distribution**
P. malabaricum (Roxb.) Benth.<br>(= *Bauhinia malabarica* Roxb.)	Bangladesh, Bhutan, Myanmar, Cambodia, India (Andhra Pradesh, Arunachal Pradesh, Assam, Bihar, Goa, Gujarat, Haryana, Himachal Pradesh, Jammu-Kashmir, Karnataka, Kerala, Madhaya, Australia (Kimberley)
P. reticulatum (DC.) Hochst.	Burkina Faso, Cameroon, Central African Republic, Chad, Ethiopia, Ghana, Ivory Coast, Mali, Niger, Nigeria, Senegal, Sudan
P. thonningii (Schum.) Milne-Redh	Angola-ISO, Benin, Botswana, Burkina Faso, Cameroon, Chad, Ethiopia, Gabon, Ghana, Guinea, Guinea Bissau, Ivory Coast, Kenya, Malawi, Mali, Mozambique, Namibia-ISO, Niger, Nigeria, Senegal, Sierra Leone, South Africa, Sudan, Tanzania, The Gambia, Togo, Uganda, Zaire, Zambia, Zimbabwe, Yemen

Table 7 The species and distribution of *Barklya* F. Muell.

**Accepted name (1)**	**Distribution**
Barklya syringifolia F. Muell.	Australia (Queensland, New South Wales)

Table 8 The species and distribution of *Lysiphyllum* (Benth.) de Wit

**Accepted names (8 in number)**	**Distribution**
L. binatum (Blanco) de Wit<br>(= *Bauhinia binata* Blanco)	Sudan, China, India, Indonesia-ISO, Java, Lesser Sunda Is, Philippines, Thailand, Australia, Papua New Guinea, Mauritius, Northern Marianas
L. carronii (F. Muell.) Pedley<br>(= *Bauhinia carronii* F. Muell.)	India, Australia (Queensland, Western Australia)
L. cunninghamii (Benth.) de Wit<br>(= *Bauhinia cunninghamii* (Benth.) Benth.)	Australia (Queensland)
L. gilvum (Bailey) Pedley<br>(= *Bauhinia gilva* (Bailey) A.S. George)	Australia (New South Wales, Northern Territory, Queensland, South Australia)
L. hookeri (F. Muell.) Pedley<br>(= *Bauhinia hookeri* F. Muell.)	India (Karnataka, Punjab, Uttar Pradesh, West Bengal), Australia

L. winitii (Craib) de Wit	Thailand
L. dewitii (K. Larsen et S.S. Larsen) Bandyop. et Ghoshal [17]	Malaysia-ISO, Sarawak
L. dipterum (Blume ex Miq.) Bandyop. et Ghoshal [17]	Brunei, China (Guizhou, Sichuan, Yunnan), Indonesia-ISO, Kalimantan, Myanmar, Thailand

Table 9 The species and distribution of *Tylosema* (Schweinf.) Torre et Hillc.

**Accepted names (5 in number)**	**Distribution**
T. argenteum (Chiov.) Brenan	Kenya, Somalia
T. esculentum (Burch.) A. Schreib.	Botswana, Namibia-ISO, South Africa
T. fassoglense (Schweinf.) Torre et Hillc.	Angola-ISO, Burundi, Ethiopia, Kenya, Malawi, Mozambique, South Africa, Sudan, Swaziland, Tanzania, Uganda, Zaire, Zambia, Zimbabwe
T. humifusum (Pic. Serm. et Roti Mich.) Brenan	Kenya, Somalia
T. angolense P. Silveira et S. Castro [49]	Southern Angola (Bié, Cuando, Cubango, Menongue, Caiundo, Capico)

Table 10 Excluded names waiting for a reclassification*

**Names (4)**	**Distribution**
B. corymbosa Roxb. ex DC.: gen. nov. ined. [16] (= *Phanera corymbosa* (Roxb. ex DC) Benth.)	China (Hainan, Guangdong), India (Punjab), Pakistan, Vietnam, Mauritius, New Zealand
B. glauca (Benth.) Benth.: gen. nov. ined. [16] (= *Phanera glauca* Benth.)	Bangladesh, Myanmar, Cambodia, China (Fujian, Guangdong, Guangxi, Guizhou, Hubei, Hunan, Jiangxi, Sichuan, Yunnan, Hong Kong), India (Arunachal Pradesh, Assam, Manipur, Meghalaya, Mizoram, Nagaland), Indonesia-ISO, Java, Laos, Malaysia-ISO, Myanmar, Peninsular Malaysia, Sumatra, Thailand, Vietnam
B. lakhonensis Gagnep.: gen. nov. ined. [16] (= *Phanera lakhonensis* (Gagnep.) A. Schmitz)	Laos, Thailand, Vietnam
B. touranensis Gagnep.: gen. nov. ined. [16] (= *Phanera touranensis* (Gagnep.) A. Schmitz)	China (Guangxi, Guizhou, Yunnan), India (Arunachal Pradesh), Laos, Myanmar, Vietnam

* Gen. nov. ined. means *gen. nova ineditus*, unpublished new genus.

References

1. *International Legume Database & Information Service (ILDIS).* Published on the Internet [http://www.ildis.org/] (accessed 12 December 2014). Cardiff: Cardiff School of Computer Science & Informatics.

2. Tu TY, Zhang DX: ***Bauhinia hekouensis* (Leguminosae, Caesalpinioideae), a new species from Yunnan, China.** *Novon* 2013, **22**:332–335.

3. Chatan W: **A new species of *Bauhinia* L. (Caesalpinioideae, Leguminosae) from Nakhon Phanom Province, Thailand.** *PhytoKeys* 2013, **26**:1–5.

4. Larsen K, Larsen SS: ***Bauhinia siamensis* (Leguminosae-Caesalpinioideae), an extraordinary new species from Thailand.** *Nat Hist Bull Siam Soc* 2002, **50**:99–104.

5. Larsen SS: ***Bauhinia wuzhengyii* (Leguminosae, Caesalpinioideae), a new Chinese species.** *Novon* 1999, **9**:526–529.

6. Queiroz LP: **New species and new combinations in *Phanera* Lour. (Caesalpinioideae: Cercideae) from the Caatinga biome.** *Neodiversity* 2006, **1**:6–10.

7. Vaz AMSF: **New combinations in *Phanera* (Leguminosae; Cercideae) from Brazil.** *Rodriguésia* 2010, **61 (Suppl.)**:S33–S40.

8. Wunderlin BP: **New combination in *Phanera* (Fabaceae).** *Phytoneuron* 2011, **19**:1–2.

9. Bandyopadhyay S, Ghoshal PP, Pathak MK: **Fifty new combinations in *Phanera* Lour. (Leguminosae: Caesalpinioideae) from paleotropical region.** *Bangladesh J Pl Taxon* 2012, **19**:55–61.

10. Wunderlin RP: **New combinations in *Schnella* (Fabaceae: Caesalpiniodeae: Cercideae).** *Phytoneuron* 2010, **49**:1–5.

11. Wunderlin RP: **Reorganization of the Cercideae (Fabaceae: Caesalpinioideae).** *Phytoneuron* 2010, **48**:1–5.

12. De Wit HCD: **A revision of Malaysian Bauhinieae.** *Reinwardtia* 1956, **3**:381–539.

13. Brenan JPM: **Leguminosae subfamily Caesalpinioideae.** In *Flora of Tropical East Africa*. Edited by Milne-Redhead E, Polhill RM. London: Crown Agents for Oversea Governments and Administrations; 1967:1–230.

14. Cayola Pérez L, Fuentes AF: ***Bauhinia tuichiensis* (Fabaceae, Cercideae), una Especie Nueva del Bosque Seco de la Región Madidi, Bolivia.** *Novon* 2012, **22**:148–151.

15. Schmidt EJD: **A new species of *Bauhinia* from southern Mozambique and the reinstatement of *Bauhinia macrantha*.** *Bothalia* 2012, **42**:44–47.

16. Mackinder BA, Clark R: **A synopsis of the Asian and Australian genus *Phanera* Lour. (Cercideae: Caesalpinioideae: Leguminosae) including 19 new combinations.** *Phytotaxa* 2014, **166**:49–68.

17. Bandyopadhyay S, Ghoshal P P.: **Two new combinations in *Lysiphyllum* (Leguminosae-Caesalpinioideae).** *Phytotaxa* 2014, **178**:288–300.

18. Darlong L, Bhattacharyya D. ***Phanera jampuiensis* (Leguminosae: Caesalpinioideae: Cercideae), a new species from Tripura, India.** *Kew Bulletin* 2014, **69**:1–6.

19. Lewis GP, Forest F: **Cercideae.** In *Legumes of the World.* Edited by Lewis G, Schrire B, Mackinder B, Lock M. Kew: The Royal Botanic Gardens; 2005:1–577.

20. George AS: ***Cercideae.*** In *Flora of Australia, Volume 12, Mimosaceae (excl. Acacia), Caesalpiniaceae.* Edited by McCarthy PM. Melbourne: ABRS/CSIRO Australia; 1998:160–167.

21. Du Puy DJ, Labat JN, Rabevohitra R, Villiers JF, Bosser J, Moat J: *The Leguminosae of Madagascar*. Kew: The Royal Botanic Gardens; 2002:1–737.

22. Thulin M: *Flora of Somalia, Volume 1, Pteridophyta; Gymnospermae; Angiospermae (Annonaceae–Fabaceae).* Kew: The Royal Botanic Gardens; 1993:1–501.

23. Larsen K, Larsen SS: ***Bauhinia* L.** In *Flora of Thailand, Volume 4 (1).* Edited by Smitinand T, Larsen K. Bangkok: The Tistr Press; 1984:4–45.

24. Wunderlin RP, Eilers RM: **Revision of *Bauhinia* subgenus *Phanera* section *Schnella* (Cercideae: Caesalpinioideae: Fabaceae).** *J Bot Research Inst Texas* 2009, **3**:619–628.

25. Vaz AMFS: **Typification of names of taxa of *Bauhinia* L. (*Leguminosae: Cercideae*) from Brazil.** *Taxon* 2011, **65:**1464–1474.

26. Fortunato RH: **Revision del Genero *Bauhinia* (Cercideae, Caesalpinioidea, Fabaceae) para la Argentina.** *Darwiniana* 1986, **27**:527–557.

27. Larsen K, Larsen SS, Vidal JE: *Flore du Cambodge du Laos et du Viê-Nam, 18*

Légumineuses-Césalpinioïdées. Paris: Muséum National D'Histoire Naturelle; 1980:1–227.
28. Wunderlin RP, Larsen K, Larsen SS: **Reorganization of the Cercideae (Fabaceae: Caesalpinioideae).** *Biol Skr* 1987, **28**:1–40.
29. Chen TC: *Bauhinia* Linn. In *Flora Reipublicae Popularis Sinicae, Tomus 39.* Edited by Wu TL, Chen PY, Wei CF, Chen TC. Beijing: Science Press; 1988:145–203.
30. Chen TC, Zhang DX, Larsen K, Larsen SS: *Bauhinia* Linnaeus. In *Flora of China, vol. 10.* Edited by Wu ZY, Raven PH, Hong DY. Beijing: Science Press & St. Louis: Missouri Botanical Garden Press; 2010:6–21.
31. Wunderlin RP: **A new species of *Bauhinia* (Leguminosae) from Peru.** *Ann Missouri Bot Gard* 1977, **64**:371–373.
32. Wunderlin RP: **Revision of the arborescent bauhinias (Fabaceae: Caesalpinioideae: Cercideae) native to Middle America.** *Ann Missouri Bot Gard* 1983, **70**:95–127.
33. Zhang DX, Chen TC: **Systematics and biogeography of *Bauhinia* L. (Leguminosae-Caesalpinioideae): I. Cladistic analysis of sect. *Lasiobema* (Korth.) Benth.** *Guihaia* 1994, **14**:11–17.
34. Burkart A: **Dos nuevas especies sudamericanas de *Bauhinia* (Legum.-Caesalp.).** *Darwiniana* 1961, **12**: 247–255.
35. Macbride JF: ***Flora of Peru*.** Publication 531, Field Museum of Natural History, Botanical Series, Vol. 13 (3-1). Chicago: Field Museum Press; 1943:1–507.
36. Schmitz A: **Nouvelle contribution à la taxonomie des Bauhinieae (Caesalpiniaceae).** *Bull Soc Roy Bot Belgique* **110** (1-2):12–16.
37. Chen TC: **New taxa of the genus *Bauhinia* L. from China.** *Guihaia* 1988, **8** (1):43–51.
38. Chen L: **New species of *Bauhinia* from China.** *J Arnold Arbor* 1938, **19**:129–133.
39. Vogel JR: **Observationes de Bauhiniis Americanis.** *Linnaea* 1839, **13**:297–315.
40. Gagnepain F: **Huit Espèces Nouvelles de *Bauhinia* D'Indochine.** *Bull Mus Natl Hist Nat sér 2*, 1952, **24**:312–316.
41. Gagnepain F: ***Bauhinia* nouveaux d'Extrême-Orient.** *Notul Syst (Paris)* 1912, **2** (6-7):168–194.
42. Gagnepain F: **Quelques espèces nouvelles; quelques synonymes.** *Notul Syst (Paris)* 1912, **2** (9):277–283.
43. Mildbraed J: **Plantae Tessmannianae peruvianae II.** *Notizbl Bot Gart Berlin-Dahlem* 1925, **9** (84):261–969.
44. Mildbraed J: **Plantae Tessmannianae peruvianae III.** *Notizbl Bot Gart Berlin-Dahlem* 1926, **9** (89):964–997.
45. Merrill ED, Metcalf FP: **New Kwangtung plants.** *Lingnan Sci J* 1937, **16** (1):77–88.
46. Zhu XY: **Nomenclatural novelties and new synonyms of Leguminosae in China.** *Biodiversity Sci* 2015, **23** (2):247–251.
47. Trethowan LA, Clark RP, Machinder BA: **A synopsis of the neotropical genus *Schnella* (Cercideae: Caesalpinioideae: Leguminosae) including 12 new combinations.** *Phytotaxa* 2015, **204**:237–252.
48. Zamora NA: **Una nueva especie de *Schnella* (Leguminosae, Caesalpinioideae: Cercideae) para Costa Rica.** *Phytoneuron* 2013, **12**:1–6.
49. Castro S, Silveira P, Coutinho AP, Figueiredo E: **Systematic studies in *Tylosema* (Leguminosae).** *Bot J Linn Soc* 2005, **147**:99–115.

50. Thulin M, Nusbaumer L, Gautier L: ***Bauhinia darainensis*** **Thulin & Nusb. (Fabaceae), a new species from northern Madagascar.** *Candollea* 2014, **69**:135–139.
