# Supplementary material for: Bilobate leaves of Bauhinia (Leguminosae, Caesalpinioideae, Cercideae) from the middle Miocene of Fujian Province, southeastern China and their biogeographic implications
Source: BMC Evol Biol. 2015 Nov 16;15:252. doi: 10.1186/s12862-015-0540-9 (PMC4647482; doi:10.1186/s12862-015-0540-9)
Supplement: Additional file 2: — The list of representative species in Fig. 2 . (PDF 141 kb) [file 12862_2015_540_MOESM2_ESM.pdf]

Additional file 2: The list of representative species in Figure 2.

(**I**) *Lysiphyllum cunninghamii* (Benth.) de Wit
(**II**) *Lysiphyllum carronii* (F. Muell.) Pedley.
(**III**) *Lysiphyllum binatum* (Blanco) de Wit
(**IV**) *Piliostigma reticulatum* (DC.) Hochst.
(**V**) *Tylosema fassoglense* (Schweinf.) Torre et Hillc.
(**VI**) *Barklya syringifolia* F. Muell.
(**VII**) *Gigasiphon humblotianum* (Baill.) Drake
(**VIII-IX**) *Bauhinia divaricata* L.
(**X**) *Bauhinia purpurea* L.
(**XI**) *Bauhinia blakeana* Dunn.
(**XII**) *Bauhinia variegata* L.
(**XIII**) *Bauhinia acuminata* L.
(**XIV**) *Bauhinia monandra* Kurz.
(**XV**) *Bauhinia aculeata* L.
(**XVI**) *Bauhinia racemosa* Lam.
(**XVII**) *Bauhinia galpinii* N.E. Br.
(**XVIII**) *Bauhinia petersiana* Bolle.
(**XIX**) *Bauhinia tomentosa* L.
(**XX**) *Bauhinia bowkeri* Harv.
(**XXI**) *Bauhinia rufescens* Lam.
(**XXII**) *Bauhinia forficata* Link.
(**XXIII**) *Bauhinia ungulata* L.
(**XXIV**) *Bauhinia grandidieri* Baill.
(**XXV**) *Bauhinia leucantha* Thulin
(**XXVI**) *Bauhinia pervilleana* Baill.
(**XXVII**) *Bauhinia somalensis* Pic. Serm. et Roti Mich.
(**XXVIII**) *Bauhinia morondavensis* Du Puy et R. Rabev.
(**XXIX**) *Bauhinia ankarafantsikae* Du Puy et R. Rabev.
(**XXX**) *Bauhinia natalensis* Hook.
(**XXXI**) *Bauhinia kalantha* Harms.
(**XXXII**) *Bauhinia mendoncae* Torre et Hillc.
(**XXXIII**) *Bauhinia urbaniana* Schinz.
(**XXXIV**) *Bauhinia madagascariensis* Desv.
(**XXXV**) *Bauhinia capuronii* Du Puy et R. Rabev.
(**XXXVI**) *Bauhinia brevicalyx* Du Puy et R. Rabev.
(**XXXVII**) *Bauhinia decandra* Du Puy et R. Rabev.
(**XXXVIII**) *Bauhinia ombrophila* Du Puy et R. Rabev.
(**XXXIX**) *Bauhinia hildebrandtii* Vatke.
(**XL**) *Bauhinia grevei* Drake
(**XLI**) *Bauhinia hagenbeckii* Harms.
(**XLII**) *Bauhinia conwayi* Rusby
(**XLIII**) *Bauhinia seminarioi* Eggers.

(**XLIV**) *Bauhinia haughtii* Wunderlin
(**XLV**) *Bauhinia subclavata* Benth.
(**XLVI**) *Bauhinia tarapotensis* Benth.
(**XLVII**) *Bauhinia longifolia* (Bong.) Steud.
(**XLVIII**) *Bauhinia urocalyx* Harms.
(**XLIX**) *Bauhinia acuruana* Moric.
(**L**) *Bauhinia cinnamomea* DC.
(**LI**) *Bauhinia brachycalyx* Ducke
(**LII**) *Bauhinia aureopunctata* Ducke
(**LIII-LIV**) *Bauhinia brachycarpa* Benth.
(**LV**) *Bauhinia hirsuta* Weinm.
(**LVI**) *Bauhinia gilesii* F. Muell. et Bailey
(**LVII**) *Bauhinia foveolata* Dalzell.
(**LVIII**) *Lasiobema scandens* (L.) de Wit
(**LIX**) *Lasiobema pulla* (Craib) A. Schmitz.
(**LX**) *Lasiobema godefroyi* (Gagnep.) comb. nov. (see Additional file 1)
(**LXI**) *Lasiobema oxysepala* (Gagnep.) comb. nov. (see Additional file 1)
(**LXII**) *Lasiobema retusa* (Roxb.) de Wit
(**LXIII**) *Lasiobema cardinale* (Gagnep.) de Wit
(**LXIV-LXXVII**) *Lasiobema championii* (Benth.) de Wit
(**LXXVIII**) *Phanera coccinea* Lour.
(**LXXIX**) *Phanera yunnanensis* (Franch.) Wunderlin
(**LXXX**) *Phanera didyma* (T.C. Chen) comb. nov. (see Additional file 1)
(**LXXXI**) *Phanera damiaoshanensis* (T.C. Chen) comb. nov. (see Additional file 1)
(**LXXXII**) *Phanera vahlii* (Wight et Arn.) Benth.
(**LXXXIII**) *Phanera pyrrhoclada* (Drake) de Wit
(**LXXXIV**) *Phanera aurea* (H. Lév.) Mackinder et R. Clark
(**LXXXV**) *Phanera carcinophylla* (Merr.) Mackinder et R. Clark
(**LXXXVI**) *Phanera lorantha* (Gagnep.) comb. nov. (see Additional file 1)
(**LXXXVII**) *Phanera nervosa* Benth.
(**LXXXVIII**) *Phanera chalcophylla* (L. Chen) Mackinder et R. Clark
(**LXXXIX**) *Phanera erythropoda* (Hayata) Mackinder et R. Clark
(**XC-XCII**) *Phanera ornata* (Kurz) Thoth.
(**XCIII**) *Phanera khasiana* (Baker) Thoth.
(**XCIV**) *Phanera audax* de Wit
(**XCV**) *Phanera paucinervata* (T.C. Chen) Mackinder et R. Clark
(**XCVI**) *Phanera lambiana* (Baker f.) de Wit
(**XCVII**) *Schnella macrostachya* Raddi
(**XCVIII**) *Schnella glabra* (Jacq.) Dugand.
(**XCIX**) *Schnella hirsutissima* (Wunderlin) Trethowan et R. Clark
(**C**) *Schnella porphyrotricha* (Harms) Wunderlin
(**CI**) *Schnella outimouta* (Aubl.) Wunderlin
(**CII**) *Schnella accrescens* (Killip et J.F. Macbr.) Trethowan et R. Clark
(**CIII**) *Bauhinia cheniae* Q. Wang et al.

(**CIV**) *Bauhinia ningmingensis* Q. Wang et al.
(**CV**) *Bauhinia larsenii* D.X. Zhang et Y.F. Chen
(**CVI**) *Bauhinia wenshanensis* H.H. Meng et Z.K. Zhou
(**CVII**) *Bauhinia nepalensis* N. Awasthi et N. Prasad
(**CVIII**) *Bauhinia* sp. 3.
(**CIX**) *Bauhinia krishnanunnii* A.K. Mathur et al.
(**CX**) *Bauhcis moranii* Calvillo-Canadell et Cevallos-Ferriz.
(**CXI**) *Bauhinia ecuadorensis* E.W. Berry
(**CXII**) *Bauhinia waylandii* R.W. Chaney
(**CXIII**) *Bauhinia siwalika* R.N. Lakh. et N. Awasthi
(**CXIV**) *Cassia rottensis* Weyland
(**CXV**) *Mimosa weberi* Schimp.
(**CXVI**) *Mimosa deperdita* Saporta
(**CXVII**) *Mimosa ayamadi* Marion
