# Supplementary material for: Bilobate leaves of Bauhinia (Leguminosae, Caesalpinioideae, Cercideae) from the middle Miocene of Fujian Province, southeastern China and their biogeographic implications
Source: BMC Evol Biol. 2015 Nov 16;15:252. doi: 10.1186/s12862-015-0540-9 (PMC4647482; doi:10.1186/s12862-015-0540-9)
Supplement: Additional file 3: — Information on voucher specimens used in this study. (DOC 177 kb) [file 12862_2015_540_MOESM3_ESM.doc]

**Additional file 3: Information on voucher specimens used in this study.**

| **Taxon** | **Number** | **Preservation state** | **Locality** | **Age** | **Repository** |
| --- | --- | --- | --- | --- | --- |
| *Barklya syringifolia* F. Muell. | K000760882, K000760883 | Herbarium | Rockhampton, Australia | Recent | K |
|  | MEL19047 |  | Queensland, Australia |  | MEL |
| *Bauhinia aculeata* L. | MA812128 | Herbarium | Peru | Recent | MA |
| *Bauhinia* sp. | BSIP36284 | Impression | Mahuadanr Valley, Palamu District, Bihar, India | Neogene | BSIP |
| *B. acuminata* L. | PE00327052, PE00327053 | Herbarium | Ledong, Hainan, China | Recent | PE |
|  | PE1650948, PE1650949 | Herbarium | Ledong, Guangdong, China | Recent | PE |
| *B. acuruana* Moric. | BR0000006411367 | Herbarium | Brazil | Recent | BR |
| *B. ankarafantsikae* Du Puy et R. Rabev. | P00090350 | Herbarium | Canton Tsaramandroso, Dct Ambato-Boéni, Madagascar | Recent | P |
| *B. aureopunctata* Ducke | S-R-8739 | Herbarium | Brazil | Recent | S |
| *B. blakeana* Dunn | PE00323321, | Herbarium | Fuzhou, Fujian, China | Recent | PE |
|  | PE[00327054](http://pe.ibcas.ac.cn/sptest/spsearchresult.aspx?x=4), PE[00327055](http://pe.ibcas.ac.cn/sptest/spsearchresult.aspx?x=5) | Herbarium | Hexian, Guangxi, China | Recent | PE |
| *B. bowkeri* Harv. | TCD0001919 | Herbarium | South Africa | Recent | TCD |
| *B. brachycalyx* Ducke | NY00003448 | Herbarium | Brazil | Recent | NY |
| *B. brachycarpa* Benth. | PE[00462941](http://pe.ibcas.ac.cn/sptest/spsearchresult.aspx?x=254), PE[00462942](http://pe.ibcas.ac.cn/sptest/spsearchresult.aspx?x=255), PE[00462943](http://pe.ibcas.ac.cn/sptest/spsearchresult.aspx?x=256), PE[00462944](http://pe.ibcas.ac.cn/sptest/spsearchresult.aspx?x=257), PE[00462945](http://pe.ibcas.ac.cn/sptest/spsearchresult.aspx?x=258) | Herbarium | Deqin, Yunnan,  China | Recent | PE |
|  | PE[00323611](http://pe.ibcas.ac.cn/sptest/spsearchresult.aspx?x=420), PE[00323612](http://pe.ibcas.ac.cn/sptest/spsearchresult.aspx?x=421), PE[00323613](http://pe.ibcas.ac.cn/sptest/spsearchresult.aspx?x=422) | Herbarium | Jinchuan, Sichuan, China | Recent | PE |
| *B. brevicalyx* Du Puy et R. Rabev. | P00131615 | Herbarium | Madagascar | Recent | P |
| *B. capuronii* Du Puy et R. Rabev. | K000417705 | Herbarium | Madagascar | Recent | K |
| *B. cheniae* Q. Wang et al. | 011656a, b, 011657, 011658, 011659, 011660, 011661, 011662, 011663, 011664, 011665, 011666, 011667, 011668, 011669, 011670, 011671, 011672, 011673, 011674, 011675 | Impression | Ningming, Guangxi, China | Possibly late Eocene or Oligocene | NHMG |
| *B. cinnamomea* DC. | F0BN001572 | Herbarium | French Guiana | Recent | F |
|  | PE[01932339](http://pe.ibcas.ac.cn/sptest/spsearchresult.aspx?x=0), PE[01932340](http://pe.ibcas.ac.cn/sptest/spsearchresult.aspx?x=1) | Herbarium | French Guiana | Recent | PE |
| *B. conwayi* Rusby  (= *B. straussiana* Harms, *B.* *tumupasensis* Rusby) | NY00003453 | Herbarium | Bolivia | Recent | NY |
| *B. decandra* Du Puy et R. Rabev. | P00090826 | Herbarium | Madagascar | Recent | P |
| *B.* *divaricata* L. | PE[01685085](http://pe.ibcas.ac.cn/sptest/spsearchresult.aspx?x=0)  PE[00463020](http://pe.ibcas.ac.cn/sptest/spsearchresult.aspx?x=1)  K000264647  US00001280 | Herbarium  Herbarium  Herbarium  Herbarium | Nicaragua  [Mexico](../../../../C:/Documents%20and%20Settings/Administrator/Local%20Settings/Application%20Data/Yodao/DeskDict/frame/20141215191217/javascript:void(0)%3B)  Haiti  Haiti | Recent  Recent  Recent  Recent | PE  K  US |
| *B.* *forficata* Link  (= *B. armata* Thunb., *B. aculeata* Vell.) | PE01684928 | Herbarium | Brazil | Recent | PE |
|  | BR0000006411503 | Herbarium | Brazil | Recent | BR |
|  | K000264656 | Herbarium | Brazil | Recent | K |
| *B. foveolata* Dalzell | K000760768, K000760769 | Herbarium | Concan, India | Recent | K |
| *B. fotana* F.M.B. Jacques et al. [this paper] | PB21577, PB21578, PB21579, PB21580, PB21581a, b, PB21582, PB21583 | Impression | Lindai Village, Zhangpu County, Zhangzhou, Fujian, China | Middle Miocene | PB |
| *B. galpinii* N.E. Br. | PE01685122 | Herbarium | Malaysia | Recent | PE |
| *B. gilesii* F. Muell. et Bailey | MEL69717 | Herbarium | Australia | Recent | MEL |
| *B. grandidieri* Baill. | P00090390 | Herbarium | Madagascar | Recent | P |
| *B. grevei* Drake | K000417700, K000417701 | Herbarium | Madagascar | Recent | K |
| *B. hagenbeckii* Harms  (*= B. hassleriana* Chodat) | BM000545082, BM000545083, BM000545084 | Herbarium | Paraguay | Recent | BM |
| *B. haughtii* Wunderlin | US00090955 | Herbarium | Ecuador | Recent | US |
| *B. hildebrandtii* Vatke | M0108430 | Herbarium | Madagascar | Recent | M |
| *B. hirsuta* Weinm.  (= *B. parvula* Gagnep.) | PE00324084 | Herbarium | Jinping, Yunnan, China | Recent | PE |
|  | PE01685090 | Herbarium | Thailand | Recent | PE |
| *B. kalantha* Harms | K000417712 | Herbarium | Tanzania | Recent | K |
| *B. larsenii* D.X. Zhang et Y.F. Chen | 45003, 45004, 45012, 45019, 011676, 011678, 011677, 011679 | Impression | Ningming, Guangxi, China | Possibly late Eocene or Oligocene | NHMG |
| *B. leucantha* Thulin | FT001368 | Herbarium | Somalia | Recent | FT |
| *B. longifolia* (Bong.) Steud. (= *B. geminata* Vogel, *B. obtusata* Vogel, *B. recurva* R.S. Cowan) | P00798628 | Herbarium | Brazil | Recent | P |
| *B. madagascariensis* Desv.  (= *B. commersonii* Decne.) | K000417697 | Herbarium | Madagascar | Recent | K |
| *B. mendoncae* Torre et Hillc. | BM000556305, BM000556313 | Herbarium | Angola | Recent | BM |
| *B. monandra* Kurz  (= *B. porosa* Boivin ex Baill., *B. punctiflora* Baker) | PE01140472 | Herbarium | Mengla, Yunnan, China | Recent | PE |
|  | K000264796 | Herbarium | French Guiana | Recent | K |
| *B. morondavensis* Du Puy et R. Rabev. | P00090367, P00090369 | Herbarium | Madagascar | Recent | P |
| *B. natalensis* Hook. | K000417676, K000417677 | Herbarium | South Africa | Recent | K |
| *B. nepalensis* N. Awasthi et N. Prasad | BSIP36501, BSIP36502 | Impression | Surai Khola beds, near Surai Khola bridge, Surai Khola, Nepal | Late Miocene–late Pleistocene | BSIP |
| *B. ningmingensis* Q Wang et al. | 011654, 011655 | Impression | Ningming, Guangxi, China | Possibly late Eocene or Oligocene | NHMG |
| *B. ombrophila* Du Puy et R. Rabev. | P00090355,  P00090356, P00090357 | Herbarium | Madagascar | Recent | P |
| *B. pervilleana* Baill. | P00131707, P00131708 | Herbarium | Madagascar | Recent | P |
| *B. petersiana* Bolle | K000417711 | Herbarium | Mozambique | Recent | K |
| *B. purpurea* L. | PE00324322 | Herbarium | Fuzhou, Fujian, China | Recent | PE |
|  | PE19501020, PE19501015 | Herbarium | Guangzhou, Guangdong, China | Recent | PE |
| *B. racemosa* Lam.  (= *B. parviflora* Vahl) | PE01932343 | Herbarium | India | Recent | PE |
| *Bauhinia ramthiensis* Antal et N. Awasthi | BSIP36924 | Impression | Right bank of upsteam of Ramthi River near Oodlabari, Darjeeling District, West Bengal, India | Middle Miocene | BSIP |
| *B. rufescens* Lam. | PE[01685116](http://pe.ibcas.ac.cn/sptest/spsearchresult.aspx?x=2),  PE[01685117](http://pe.ibcas.ac.cn/sptest/spsearchresult.aspx?x=1),  PE[01685118](http://pe.ibcas.ac.cn/sptest/spsearchresult.aspx?x=0) | Herbarium | Ghana | Recent | PE |
| *B. seminarioi* Eggers | F0BN001593 | Herbarium | Ecuador | Recent | F |
| *B. siwalika* R.N. Lakh. et N. Awasthi | BSIP35344 | Impression | Siwalik, Bhikhnathoree, West Champaran District, Bihar; Cherrapunji, West Khasi Hills District, Meghalaya, India | Middle Miocene–middle Pleistocene | BSIP |
| *B. somalensis* Pic. Serm. et Roti Mich. | K000417715 | Herbarium | Somalia | Recent | K |
| *B. subclavata* Benth. | K000056820,  K000056821, K000056823 | Herbarium | Brazil | Recent | K |
| *B. tarapotensis* Benth.  (= *B. amplifolia* Ducke) | K000201023,  K000201025 | Herbarium | Peru | Recent | K |
|  | NY00003499 | Herbarium | Peru | Recent | NY |
| *B. tomentosa* L. | PE[01140455](http://pe.ibcas.ac.cn/sptest/spsearchresult.aspx?x=0) | Herbarium | Mengla, Yunnan, China | Recent | PE |
|  | PE[01685097](http://pe.ibcas.ac.cn/sptest/spsearchresult.aspx?x=1) | Herbarium | India | Recent | PE |
|  | PE[01685138](http://pe.ibcas.ac.cn/sptest/spsearchresult.aspx?x=2) | Herbarium | Indonesia | Recent | PE |
| *B. ungulata* L.  (= *B. macrostachya* Benth.) | PE[01685093](http://pe.ibcas.ac.cn/sptest/spsearchresult.aspx?x=3), PE[01685093](http://pe.ibcas.ac.cn/sptest/spsearchresult.aspx?x=3) | Herbarium | Brazil | Recent | PE |
|  | PE[01685366](http://pe.ibcas.ac.cn/sptest/spsearchresult.aspx?x=1), PE[01685367](http://pe.ibcas.ac.cn/sptest/spsearchresult.aspx?x=2) | Herbarium | Bolivia | Recent | PE |
|  | MA601287, MA601288 | Herbarium | Mexico | Recent | MA |
|  | MA666151,  MA666152,  MA666153 | Herbarium | Colombia | Recent | MA |
| *B. ungulatoides* Y.X. Lin et al. sp. nov. [this paper] | PB21584a, b, c, d, PB21585, PB21586 | Compression, impression, slide | Lindai Village, Zhangpu County, Zhangzhou, Fujian, China | Middle Miocene | PB |
| *B. urbaniana* Schinz | K000417686 | Herbarium | South Africa | Recent | K |
| *B. urocalyx* Harms | F0BN001625 | Herbarium | Brazil | Recent | F |
| *B. variegata* L. | PE[00324348](http://pe.ibcas.ac.cn/sptest/spsearchresult.aspx?x=2) | Herbarium | Xiamen, Fujian, China | Recent | PE |
|  | PE[00324351](http://pe.ibcas.ac.cn/sptest/spsearchresult.aspx?x=3) | Herbarium | Fuzhou, Fujian, China | Recent | PE |
|  | PE[00324361](http://pe.ibcas.ac.cn/sptest/spsearchresult.aspx?x=13),  PE[00324362](http://pe.ibcas.ac.cn/sptest/spsearchresult.aspx?x=14) | Herbarium | Guangzhou, Guangdong, China | Recent | PE |
| *Cassia bauhinioides* A. Gray | k000789217  US00001402 | Herbarium  Herbarium | United States  United States | Recent  Recent | K  US |
| *Gigasiphon humblotianum* (Baill.) Drake  (= *Bauhinia humblotiana* Baill.) | MO-718043 | Herbarium | Madagascar | Recent | MO |
|  | P00131659, P00131660, P00131661 | Herbarium | Madagascar | Recent | P |
| *Lasiobema*  *cardinale* (Pierre ex Gagnep.) de Wit  (= *Bauhinia cardinalis* Pierre ex Gagnep., *B. dolichobotrys* Merr.) | P00798455,  P00798456, P00798457,  P00798458  P00798459 | Herbarium | Vietnam | Recent | P |
| *Lasiobema*  *championii* (Benth.) de Wit  (= *Phanera championii* Benth., *Bauhinia* *championii* (Benth.) Benth., *B. esquirolii* Gagnep.) | PE00323810 | Herbarium | Xiamen, Fujian, China | Recent | PE |
|  | PE[00323811](http://pe.ibcas.ac.cn/sptest/spsearchresult.aspx?x=12), PE[00327867](http://pe.ibcas.ac.cn/sptest/spsearchresult.aspx?x=60), PE[00327865](http://pe.ibcas.ac.cn/sptest/spsearchresult.aspx?x=58) | Herbarium | Pinghe, Fujian, China | Recent | PE |
|  | PE[00323812](http://pe.ibcas.ac.cn/sptest/spsearchresult.aspx?x=13) | Herbarium | Dehua,Fujian, China | Recent | PE |
|  | PE[00323940](http://pe.ibcas.ac.cn/sptest/spsearchresult.aspx?x=135), PE[00323941](http://pe.ibcas.ac.cn/sptest/spsearchresult.aspx?x=136), PE[00323942](http://pe.ibcas.ac.cn/sptest/spsearchresult.aspx?x=137), PE[00323943](http://pe.ibcas.ac.cn/sptest/spsearchresult.aspx?x=138), PE[00323944](http://pe.ibcas.ac.cn/sptest/spsearchresult.aspx?x=139), PE[00323945](http://pe.ibcas.ac.cn/sptest/spsearchresult.aspx?x=140) | Herbarium | Nanjing, Fujian, China | Recent | PE |
|  | PE[00323853](http://pe.ibcas.ac.cn/sptest/spsearchresult.aspx?x=69), PE[00323854](http://pe.ibcas.ac.cn/sptest/spsearchresult.aspx?x=70), PE[00323855](http://pe.ibcas.ac.cn/sptest/spsearchresult.aspx?x=71) | Herbarium | Wengyuan, Guangdong, China | Recent | PE |
| *Lasiobema*  *godefroyi* (Gagnep.) com. nov. [this paper]  Basionym: *Bauhinia godefroyi* Gagnep. in *Notul Syst (Paris)* 2: 278. 1912 | P00798543, P00798544 | Herbarium | Cambodia | Recent | P |
| *Lasiobema*  *oxysepala* (Gagnep.) com. nov. [this paper]  Basionym: *Bauhinia oxysepala* Gagnep. in *Notul Syst (Paris)* 2: 176. 1912 | P00798528,  P00798529 | Herbarium | Vietnam | Recent | P |
| *Lasiobema*  *pulla* (Craib) A. Schmitz  (= *Bauhinia pulla* Craib) | K000760812 | Herbarium | Thailand | Recent | K |
|  | TCD0016408, TCD0016421 | Herbarium | Thailand | Recent | TCD |
| *L.* *retusa* (Roxb.) de Wit | E00317659, E00317660 | Herbarium | India | Recent | E |
| *L.* *scandens* (L.) de Wit | PE[00327083](http://pe.ibcas.ac.cn/sptest/spsearchresult.aspx?x=0), PE[00324471](http://pe.ibcas.ac.cn/sptest/spsearchresult.aspx?x=4) | Herbarium | Jianfeng, Hainan, China | Recent | PE |
|  | PE[00324469](http://pe.ibcas.ac.cn/sptest/spsearchresult.aspx?x=2), PE[00324472](http://pe.ibcas.ac.cn/sptest/spsearchresult.aspx?x=5) | Herbarium | Dongfang, Hainan, China | Recent | PE |
| *Lysiphyllum binatum* (Blanco) de Wit  (= *Bauhinia binata* Blanco) | MO-149924 | Herbarium | The Philippines | Recent | MO |
| *L. carronii* (F. Muell.) Pedley (= *Bauhinia carronii* F. Muell.) | K000760895, K000760897 | Herbarium | Australia | Recent | K |
| *L. cunninghamii* (Benth.) de Wit (= *Bauhinia cunninghamii* (Benth.) Benth.) | BRI-AQ0336264, BRI-AQ0024992 | Herbarium | Queensland, Australia | Recent | BRI |
|  | K000760890, K000760900, K000780143, K000760889, K000760898 | Herbarium | Australia | Recent | K |
| *Mimosa albida* Humb. et Bonpl. ex Willd. | F0BN001295  US00000791 | Herbarium  Herbarium | Peru  Mexico | Recent  Recent | P  US |
| *M. ceratonia* L. | MA601633 | Herbarium | Mexico | Recent | MA |
| *M.* *spruceana* Benth. | G00371570  G00371606 | Herbarium | Brazil | Recent | G |
| *Phanera audax* de Wit  (= *Bauhinia calycina* Gagnep.) | P00798464, P00798465 | Herbarium | Cambodia | Recent | P |
| *P. aurea* (H. Lév.) Mackinder et R. Clark | PE[00323310](http://pe.ibcas.ac.cn/sptest/spsearchresult.aspx?x=6), PE[00323311](http://pe.ibcas.ac.cn/sptest/spsearchresult.aspx?x=7), PE[00323312](http://pe.ibcas.ac.cn/sptest/spsearchresult.aspx?x=8) | Herbarium | Jinghong, Yunnan, China | Recent | PE |
| *P. carcinophylla* (Merr.) Mackinder et R. Clark | A00059772 | Herbarium | Vietnam | Recent | A |
| *P. chalcophylla* (L. Chen) Mackinder et R. Clark | PE[00323866](http://pe.ibcas.ac.cn/sptest/spsearchresult.aspx?x=0), PE[00020914](http://pe.ibcas.ac.cn/sptest/spsearchresult.aspx?x=1) | Herbarium | Yongshan, Yunnan, China | Recent | PE |
| *P. coccinea* Lour. | PE00327067 | Herbarium | Pingbian, Yunnan, China | Recent | PE |
|  | P00798553 | Herbarium | Vietnam | Recent | P |
| *P. damiaoshanensis* (T.C. Chen) comb. nov. [this paper]  Basionym: *Bauhinia damiaoshanensis* T.C. Chen in *Guihaia* 8: 49. 1988 | PE01965846 | Herbarium | Napo, Guangxi, China | Recent | PE |
|  | K000760719 | Herbarium | Damiaoshan, Guangxi, China | Recent | K |
| *P. didyma* (L. Chen) comb. nov. [this paper]  Basionym: *Bauhinia didyma* L. Chen in *J Arnold Arbor* 19: 131. 1938 | PE[00323954](http://pe.ibcas.ac.cn/sptest/spsearchresult.aspx?x=0), PE[00323955](http://pe.ibcas.ac.cn/sptest/spsearchresult.aspx?x=1) | Herbarium | Yangchun, Guangdong, China | Recent | PE |
| *P. erythropoda* (Hayata) Mackinder et R. Clark | PE[00323968](http://pe.ibcas.ac.cn/sptest/spsearchresult.aspx?x=0), PE[00323972](http://pe.ibcas.ac.cn/sptest/spsearchresult.aspx?x=4) | Herbarium | Baoting, Hainan, China | Recent | PE |
| *P. khasiana* (Baker) Thoth. | PE[00461138](http://pe.ibcas.ac.cn/sptest/spsearchresult.aspx?x=0), PE[00461139](http://pe.ibcas.ac.cn/sptest/spsearchresult.aspx?x=1), PE[00324085](http://pe.ibcas.ac.cn/sptest/spsearchresult.aspx?x=2), PE[00324297](http://pe.ibcas.ac.cn/sptest/spsearchresult.aspx?x=3) | Herbarium | Sanya, Hainan, China | Recent | PE |
| *P. lambiana* (Baker f.) de Wit | L0056518 | Herbarium | Malaysia | Recent | L |
| *P. lorantha* (Pierre ex Gagnep.) comb. nov. [this paper]  Basionym: *Bauhinia lorantha* Pierre ex Gagnep. in *Notul Syst (Paris)* 2: 175. 1912 | P00798534, P00798535 | Herbarium | Laos | Recent | P |
| *P. nervosa* Benth. | K000760781 | Herbarium | India | Recent | K |
| *P. ornata* (Kurz) Thoth. | PE[00323405](http://pe.ibcas.ac.cn/sptest/spsearchresult.aspx?x=0), PE[00323406](http://pe.ibcas.ac.cn/sptest/spsearchresult.aspx?x=0), PE[00323407](http://pe.ibcas.ac.cn/sptest/spsearchresult.aspx?x=0), PE[00324314](http://pe.ibcas.ac.cn/sptest/spsearchresult.aspx?x=7) | Herbarium | Chengmai, Hainan, China | Recent | PE |
| *P. paucinervata* (T.C. Chen) X.Y. Zhu | PE[00327075](http://pe.ibcas.ac.cn/sptest/spsearchresult.aspx?x=0), PE[00327076](http://pe.ibcas.ac.cn/sptest/spsearchresult.aspx?x=1) | Herbarium | Pingxiang, Guangxi, China | Recent | PE |
| *P. pyrrhoclada* (Drake) de Wit | PE[00324321](http://pe.ibcas.ac.cn/sptest/spsearchresult.aspx?x=0), PE[00324464](http://pe.ibcas.ac.cn/sptest/spsearchresult.aspx?x=2), PE[00324465](http://pe.ibcas.ac.cn/sptest/spsearchresult.aspx?x=2), PE[00324466](http://pe.ibcas.ac.cn/sptest/spsearchresult.aspx?x=2) | Herbarium | Wangning, Hainan, China | Recent | PE |
| *P. vahlii* (Wight et Arn.) Benth. | E00174587, E00174588, E00174589, | Herbarium | India | Recent | E |
| *P. yunnanensis* (Franch.) Wunderlin  (= *Bauhinia yunnanensis* Franch., *P. collettii* Thoth.) | PE[00324440](http://pe.ibcas.ac.cn/sptest/spsearchresult.aspx?x=29), PE[00324442](http://pe.ibcas.ac.cn/sptest/spsearchresult.aspx?x=31), PE[00324443](http://pe.ibcas.ac.cn/sptest/spsearchresult.aspx?x=32), PE[00324444](http://pe.ibcas.ac.cn/sptest/spsearchresult.aspx?x=33) | Herbarium | Binchuan, Yunan, China | Recent | PE |
| *Piliostigma reticulatum* (DC.) Hochst. | P00374760,  P00374761,  P00374762, P00374763, P00374764, | Herbarium | Ethiopia | Recent | P |
| *Schnella accrescens* (Killip et J.F. Macbr.) Trethowan et R. Clark | US00001251 | Herbarium | Peru | Recent | US |
|  | F0042815F, F0042816F | Herbarium | Peru | Recent | F |
| *S. glabra* (Jacq.) Dugand. | MO-714949 | Herbarium | Peru | Recent | MO |
| *S. hirsutissima* (Wunderlin) Trethowan et R. Clark | NY00003474 | Herbarium | Peru | Recent | NY |
|  | K000264809 | Herbarium | Peru | Recent | K |
| *S. macrostachya* Raddi  (= *Bauhinia radiata* Vell., *Phanera radiata* (Vell.) Vaz) | G00367730 | Herbarium | Brazil | Recent | G |
|  | P00798606 | Herbarium | Brazil | Recent | P |
|  | K000264702 | Herbarium | Brazil | Recent | K |
| *S. outimouta* (Aubl.) Wunderlin | BM000603760 | Herbarium | Guyana | Recent | BM |
| *S. porphyrotricha* (Harms) Wunderlin | F0BN001605, F0042788F | Herbarium | Peru | Recent | F |
| *Tylosema fassoglense* (Schweinf.) Torre et Hillc. | WAG0003825, WAG0003826 | Herbarium | Ethiopia | Recent | WAG |

***Notes:*** A: Harvard University, Cambridge; BM: The Natural History Museum, London; BR: National Botanic Garden of Belgium, Meise; BRI: Queensland Herbarium, Brisbane; BSIP: Birbal Sahni Institute of Palaeobotany, Lucknow; E: Royal Botanic Garden Edinburgh, Edinburgh; F: Field Museum of Natural History, Chicago; FT: Centro Studi Erbario Tropicale Università degli Studi di Firenze, Firenze; G: Conservatoire et Jardin Botaniques de la Ville de Genève, Genève; K: Royal Botanic Gardens, Kew; L: Nationaal Herbarium Nederland, Leiden University Branch, Leiden; M: Botanische Staatssammlung München, München; MA: Real Jardín Botánico, Madrid; MEL: National Herbarium of Victoria, Melbourne; MO: Missouri Botanical Garden, Missouri; NY: The New York Botanical Garden, Bronx; NHMG: Natural History Museum of Guangxi, Nanning; P, Muséum National d'Histoire Naturelle, Paris; PB: Nanjing Institute of Geology and Palaeontology, Nanjing; PE: the Chinese National Herbarium, Beijing; S: Swedish Museum of Natural History, Stockholm; TCD: Trinity College, Dublin; US: Smithsonian Institution, Washington; WAG: Wageningen University, Wageningen (see *Index Herbariorum* [http://sweetgum.nybg.org/ih/]).
