# Supplementary material for: Bilobate leaves of Bauhinia (Leguminosae, Caesalpinioideae, Cercideae) from the middle Miocene of Fujian Province, southeastern China and their biogeographic implications
Source: BMC Evol Biol. 2015 Nov 16;15:252. doi: 10.1186/s12862-015-0540-9 (PMC4647482; doi:10.1186/s12862-015-0540-9)
Supplement: Additional file 4: — Morphological analyses of leaves from illustrated species in Bauhinia and its allies. (PDF 158 kb) [file 12862_2015_540_MOESM4_ESM.pdf]

Additional file 4: Morphological analyses of leaves from illustrated species in *Bauhinia* L. s.s. and its allies.

Illustrated species	Length/(Width/2)	Dissection index	Sinus (˚)
(I) *Lysiphyllum cunninghamii* (Benth.) de Wit	1.390526316	1.422142123	27
(II) *Lysiphyllum carronii* (F. Muell.) Pedley.	1.453313477	1.455869709	23
(III) *Lysiphyllum binatum* (Blanco) de Wit	1.420794507	1.462178776	16
(IV) *Piliostigma reticulatum* (DC.) Hochst.	1.702111755	1.120644981	82
(V) *Tylosema fassoglense* (Schweinf.) Torre et Hillc.	1.760935417	1.198024958	76
(VI) *Barklya syringifolia* F. Muell.	2.100603136	1.183297852	0
(VII) *Gigasiphon humblotianum* (Baill.) Drake	2.571577629	1.180837907	0
(VIII) *Bauhinia divaricata* L.	2.333749688	1.314875973	83
(IX) *Bauhinia divaricata* L. *var. angustiloba*	10.06901763	2.928960219	49
(X) *Bauhinia purpurea* L.	1.85092156	1.145731144	29
(XI) *Bauhinia blakeana* Dunn.	2.063883688	1.152293321	37
(XII) *Bauhinia variegata* L.	1.75627738	1.188713325	48
(XIII) *Bauhinia acuminata* L.	2.036238245	1.216719891	50
(XIV) *Bauhinia monandra* Kurz.	1.771466796	1.176798317	20
(XV) *Bauhinia aculeata* L.	1.894640283	1.101067724	57
(XVI) *Bauhinia racemosa* Lam.	1.44115136	1.122525844	64
(XVII) *Bauhinia galpinii* N. E. Br.	1.326474167	1.096264087	103
(XVIII) *Bauhinia petersiana* Bolle.	1.52171832	1.126108444	66
(XIX) *Bauhinia tomentosa* L.	1.602031515	1.147735473	34
(XX) *Bauhinia bowkeri* Harv.	1.896178803	1.246057946	15
(XXI) *Bauhinia rufescens* Lam.	1.428477258	1.247155922	18
(XXII) *Bauhinia forficata* Link.	2.913998453	1.578595384	21
(XXIII) *Bauhinia ungulata* L.	3.36646786	1.490419631	18
(XXIV) *Bauhinia grandidieri* Baill.	1.816265589	1.477941417	36
(XXV) *Bauhinia leucantha* Thulin	1.550669216	1.432348467	18
(XXVI) *Bauhinia pervilleana* Baill.	2.296497913	1.625772022	29
(XXVII) *Bauhinia somalensis* Pic. Serm. et Roti Mich.	1.311537578	1.40206531	10
(XXVIII) *Bauhinia morondavensis* Du Puy et R. Rabev.	1.642327785	1.481242817	17
(XXIX) *Bauhinia ankarafantsikae* Du Puy et R. Rabev.	1.426383454	1.459583272	10
(XXX) *Bauhinia natalensis* Hook.	1.87653639	1.554126765	19
(XXXI) *Bauhinia kalantha* Harms.	1.873561662	1.52958996	18
(XXXII) *Bauhinia mendoncae* Torre et Hillc.	1.662425785	1.34336794	59
(XXXIII) *Bauhinia urbaniana* Schinz.	1.386083289	1.384772659	57
(XXXIV) *Bauhinia madagascariensis* Desv.	2.04895371	1.394605224	12
(XXXV) *Bauhinia capuronii* Du Puy et R. Rabev.	1.622621107	1.262700457	24
(XXXVI) *Bauhinia brevicalyx* Du Puy et R. Rabev.	1.973128359	1.184925414	25
(XXXVII) *Bauhinia decandra* Du Puy et R. Rabev.	2.327937027	1.354055303	26
(XXXVIII) *Bauhinia ombrophila* Du Puy et R. Rabev.	2.636061613	1.349307427	27
(XXXIX) *Bauhinia hildebrandtii* Vatke.	2.86194747	1.707098488	30
(XL) *Bauhinia grevei* Drake	5.237610005	1.240014414	0
(XLI) *Bauhinia hagenbeckii* Harms.	2.382723314	1.744291197	13
(XLII) *Bauhinia conwayi* Rusby	3.493349958	1.663219833	58
(XLIII) *Bauhinia seminarioi* Eggers.	2.043022175	1.143838883	49
(XLIV) *Bauhinia haughtii* Wunderlin	1.809564684	1.271879054	24
(XLV) *Bauhinia subclavata* Benth.	1.746091842	1.074715664	80
(XLVI) *Bauhinia tarapotensis* Benth.	2.86848249	1.328392499	26
(XLVII) *Bauhinia longifolia* (Bong.) Steud.	3.821296979	1.374538643	27
(XLVIII) *Bauhinia urocalyx* Harms.	2.57336676	1.48510645	9
(XLIX) *Bauhinia acuruana* Moric.	3.504038772	1.07715308	0
(L) *Bauhinia cinnamomea* DC.	4.674227799	1.166359657	0
(LI) *Bauhinia brachycalyx* Ducke.	6.735950045	1.370061994	0
(LII) *Bauhinia aureopunctata* Ducke.	4.021706462	1.141669858	0
(LIII) *Bauhinia brachycarpa* Benth.	1.685318342	1.290400072	20
(LIV) *Bauhinia brachycarpa* Benth. 2	1.601647059	1.276447383	23
(LV) *Bauhinia hirsuta* Weinm.	1.687217329	1.073500437	91
(LVI) *Bauhinia gilesii* F. Muell. et Bailey	1.53104685	1.07642155	114
(LVII) *Bauhinia foveolata* Dalzell.	1.945306878	1.11523919	83
(LVIII) *Lasiobema scandens* (L.) de Wit.	2.046022202	1.433927685	80
(LIX) *Lasiobema pulla* (Craib) A. Schmitz.	2.2896196	1.41725443	8
(LX) *Lasiobema godefroyi* (Gagnep.) comb. nov.	3.008200923	1.562192639	42
(LXI) *Lasiobema oxysepala* (Gagnep.) comb. nov.	2.499788464	1.425336948	9
(LXII) *Lasiobema retusa* (Roxb.) de Wit	1.457056792	1.079755029	58
(LXIII) *Lasiobema cardinale* (Gagnep.) de Wit	3.439170665	1.082568887	0
(LXIV) *Lasiobema championii* (Benth.) de Wit	2.590323336	1.595092742	65

(LXV) *Lasiobema championii* (Benth.) de Wit 2	2.803588749	1.340885981	79
(LXVI) *Lasiobema championii* (Benth.) de Wit	2.526243515	1.112501616	93
(LXVII) *Lasiobema championii* (Benth.) de Wit	4.022717885	1.202075349	79
(LXVIII) *Lasiobema championii* (Benth.) de Wit	1.310951526	1.283783015	105
(LXIX) *Lasiobema championii* (Benth.) de Wit 3	2.196465428	1.043018334	125
(LXX) *Lasiobema championii* (Benth.) de Wit	2.230362753	1.053568709	55
(LXXI) *Lasiobema championii* (Benth.) de Wit	2.875321337	1.208830303	9
(LXXII) *Lasiobema championii* (Benth.) de Wit	2.550763106	1.164496628	6
(LXXIII) *Lasiobema championii* (Benth.) de Wit	3.077537233	1.063976965	0
(LXXIV) *Lasiobema championii* (Benth.) de Wit	3.281515581	1.065804842	0
(LXXV) *Lasiobema championii* (Benth.) de Wit	2.99983585	1.106926345	0
(LXXVI) *Lasiobema championii* (Benth.) de Wit	3.223680051	1.18719536	0
(LXXVII) *Lasiobema championii* (Benth.) de Wit 4	5.520984081	1.203703764	0
(LXXVIII) *Phanera coccinea* Lour.	2.6416615	1.175166964	36
(LXXIX) *Phanera yunnanensis* (Franch.) Wunderlin	1.600292991	1.462270378	42
(LXXX) *Phanera didyma* (T.C. Chen) comb. nov.	1.58893617	1.491010827	30
(LXXXI) *Phanera damiaoshanensis* (T.C. Chen) comb. nov.	1.640700483	1.112766479	52
(LXXXII) *Phanera vahlii* (Wight et Arn.) Benth.	1.812674321	1.29708095	25
(LXXXIII) *Phanera pyrrhoclada* (Drake) de Wit	2.027236278	1.25574082	41
(LXXXIV) *Phanera aurea* (H. Lév.) Mackinder et R. Clark	1.971048417	1.181548482	61
(LXXXV) *Phanera carcinophylla* (Merr.) Mackinder et R. Clark	2.708635266	1.796723255	11
(LXXXVI) *Phanera lorantha* (Gagnep.) comb. nov.	2.645385588	1.533627344	25
(LXXXVII) *Phanera nervosa* Benth.	2.563099944	1.327791619	16
(LXXXVIII) *Phanera chalcophylla* (L. Chen) Mackinder et R. Clark	2.543216144	1.288910849	18
(LXXXIX) *Phanera erythropoda* (Hayata) Mackinder et R. Clark	2.367759146	1.284280998	72
(XC) *Phanera ornata* (Kurz) Thoth.	2.227469403	1.27642541	38
(XCI) *Phanera ornata* (Kurz) Thoth.	2.337832576	1.240602529	52
(XCII) *Phanera ornata* (Kurz) Thoth.	2.890658831	1.338747536	21
(XCIII) *Phanera khasiana* (Baker) Thoth.	2.364194164	1.110460123	54
(XCIV) *Phanera audax* de Wit	2.905351067	1.090082103	0
(XCV) *Phanera paucinervata* (T.C. Chen) Mackinder et R. Clark	4.912457912	1.195699502	0
(XCVI) *Phanera lambiana* (Baker f.) de Wit	8.497082228	1.482668586	0
(XCVII) *Schnella macrostachya* Raddi	2.035714286	1.313913407	20
(XCVIII) *Schnella glabra* (Jacq.) Dugand.	3.245676418	1.64832388	25
(XCIX) *Schnella hirsutissima* (Wunderlin) comb. nov.	3.224673203	1.522063284	23
(C) *Schnella porphyrotricha* (Harms) Wunderlin	3.258222297	1.612518789	23
(CI) *Schnella outimouta* (Aubl.) Wunderlin	1.940665577	1.68387217	4
(CII) *Schnella accrescens* (Killip et J.F. Macbr.) comb. nov.	1.966895483	1.160983184	0
(CIII) *Bauhinia cheniae* Q. Wang et al.	1.804551539	1.423074557	14
(CIV) *Bauhinia ningmingensis* Q. Wang et al.	1.803816951	1.474262244	18
(CV) *Bauhinia larsenii* D.X. Zhang et Y.F. Chen	2.083343684	1.320524848	23
(CVI) *Bauhinia wenshanensis* H.H. Meng et Z. K. Zhou	2.780898876	1.628624849	14
(CVII) *Bauhinia nepalensis* N. Awasthi et N. Prasad	1.862789445	1.193115821	48
(CVIII) *Bauhinia* sp. 3.	1.840280732	1.286506433	27
(CIX) *Bauhinia krishnanunnii* A.K. Mathur et al.	2.745306551	1.20270523	120
(CX) *Bauhcis moranii* Calvillo-Canadell et Cevallos-Ferriz.	1.408874952	1.078738715	84
(CXI) *Bauhinia ecuadorensis* E.W. Berry	1.426835197	1.552111748	26
(CXII) *Bauhinia waylandii* R.W. Chaney	1.792042146	1.514580638	45
(CXIII) *Bauhinia siwalika* R.N. Lakh. et N. Awasthi	1.525610002	1.110798368	24
(CXIV) *Cassia rottensis* H. Weyland	1.981253145	1.508382471	25
(CXV) *Mimosa weberi* Schimper	3.11079865	1.749821789	25
(CXVI) *Mimosa deperdita* Saporta	3.717032967	1.867944805	61
(CXVII) *Mimosa ayamadi* Marion	3.245661157	1.860393288	81
(This paper) *Bauhinia fotana* F.M.B. Jacques, G.L. Shi et Z.K. Zhou	2.329402336	1.322199073	45
(This paper) *Bauhinia ungulatoides* Y.X. Lin et al., sp. nov.	3.438892529	1.388113989	25

Illustrated species	Average value (DI)
Lysiphyllum (Korth.) Miq.	1.446730203
Piliostigma Hochst.	1.120644981
Tylosema (Schweinf.) Torre et Hillc	1.198024958
Barklya F. Muell.	1.183297852
Gigasiphon Drake	1.180837907
Lasiobema (Korth.) Miq.	1.231644779
Phanera Lour.	1.31271604
Schnella Raddi	1.490279119
Bauhinia s.s. outside America	1.349882859
Bauhinia s.s. in America	1.320102234
Bauhinia fotana F.M.B. Jacques, G.L. Shi et Z.K. Zhou	1.322199073
Bauhinia ungulatoides Y.X. Lin et al., sp. nov.	1.388113989
